# Computational Development of Transmission-Blocking Vaccine Candidates Based on Fused Antigens of Pre- and Post-fertilization Gametocytes Against *Plasmodium falciparum*

**DOI:** 10.1177/11779322241306215

**Published:** 2025-03-03

**Authors:** Matthew A. Adeleke

**Affiliations:** Discipline of Genetics, School of Life Sciences, College of Agriculture, Engineering and Sciences, University of KwaZulu-Natal, Durban, South Africa

**Keywords:** fusion antigen, immunoinformatics, malaria, mosquitoes, transmission-blocking

## Abstract

*Plasmodium falciparum* is the most fatal species of malaria parasites in humans. Attempts at developing vaccines against the malaria parasites have not been very successful even after the approval of the RTS, S/AS01 vaccine. There is a continuous need for more effective vaccines including sexual-stage antigens that could block the transmission of malaria parasites between mosquitoes and humans. Low immunogenicity, expression, and stability are some of the challenges of transmission-blocking vaccine (TBV). This study was designed to computationally identify TBV candidates based on fused antigens by combining highly antigenic peptides from prefertilization (Pfs230, Pfs48/45) and postfertilization (Pfs25, Pfs28) gametocytes. The peptides were selected based on their antigenicity, nonallergenicity, and lack of similarity with the human proteome. Two fused antigens vaccine candidates (FAVCs) were constructed using Flagellin *Salmonella enterica* (FAVC-FSE) and Cholera toxin B (FAVC-CTB) as adjuvants. The constructs were evaluated for their physicochemical properties, structural stability, immunogenicity, and potential to elicit cross-protection across multiple *Plasmodium* species. The results yielded antigenic peptides, with antigenicity scores between 0.7589 and 1.1821. The structural analysis of FAVC-FSE and FAVC-CTB showed a Z-score of -6.70 and -4.79, a Ramachandran plot of 96.94% and 94.86% with overall quality of 94.20% and 89.85%, respectively. The FAVCs contained CD8^+^, CD4^+^, and linear B-cell epitopes with antigenicity scores between 1.2089 and 2.8623, 0.5663 and 2.4132, and 1.5196 and 2.2212, respectively. Each FAVC generated 6 conformational B-cells. High population coverage values were recorded for the FAVCs. The ability of the FAVCs to trigger immune response was evaluated through an in silico immune stimulation. The low-binding interaction energy that resulted from molecular docking and dynamics simulations showed a strong affinity of FAVCs to Toll-like receptor 5 (TLR5). The results indicate that the FAVC-FSE vaccine candidate is more promising to interrupt *P falciparum* transmission and provides a baseline for experimental validation.

## Introduction

According to the World Malaria Report 2023, 249 million malaria cases and 608 000 deaths were reported for the year 2022.^1^ About 247 million malaria cases were reported in 2021 in 84 endemic countries predominantly from Africa.^2^ In 2019, there were 229 million cases and 409 000 deaths with 94% of the deaths reported in sub-Saharan Africa with the majority of the deaths occurring with children under the age of 5.^3^ So, malaria remains a global health challenge despite different intervention strategies. Malaria infection is caused by the genus *Plasmodium*. The species that infect humans include *Plasmodium falciparum, Plasmodium vivax, Plasmodium ovale, Plasmodium malariae, and Plasmodium knowlesi* of which *P falciparum* causes the highest infection.^4^ This parasite has a complex lifecycle that makes its control a challenge, particularly through vaccination.

Following a mosquito bite, infection is established in the liver by sporozoites, which then move into blood cells to form the second stage of the infection by merozoites. This leads to the recognition of the parasites by various receptors of innate immune response.^4^ It is important to understand the host immune response to *Plasmodium* infection to ensure adequate induction and maintenance of antibodies to develop a vaccine with efficacy.

Some of the major prevention and control strategies include indoor residual spraying, insecticide-treated nets, and the use of antimalarial drugs but resistance to insecticides and antimalarials limits their effectiveness.^5^ There has been a long journey toward malaria vaccine development and the RTS, S/AS01 vaccine was approved in 2021 by the World Health Organization^3^ and reported that the RTS, S/AS01 vaccine was effective against severe malaria, with a 30% efficacy against severe malaria and a significant 21% drop in hospitalization. Another licensed malaria vaccine in April 2023 is R21/Matrix-M by the Food and Drugs Authority in Ghana to be used for children 5 to 36 months old.^6^ This vaccine was then recommended in October 2023 by the World Health Organization and later disqualified in December 2023 by the same organization.^7^ While some levels of success have been recorded with the malaria vaccine, it is crucial to sustain the efforts toward improving the current vaccine and developing new ones. There should be continued efforts in searching for more promising vaccine candidates.

Discovery of novel antigens and improvement of current vaccine candidates are being targeted at the 3 stages of malaria parasite development. These include preerythrocytic, asexual blood, and mosquito sexual stages.^8^ To break the transmission of malaria parasites from mosquitoes to humans, attention is being given to the sexual stage of the parasite’s development. Hence the need to target transmission-blocking vaccine (TBV) candidates. For *P falciparum*, the important TBV candidates include prefertilization (Pfs230 and Pfs48/45) and postfertilization (Pfs25 and Pfs28) antigens.^8,9^ The prefertilization proteins, Pfs230 and Pfs48/45 help male gametes in the recognition and attachment to female gametes including the formation of ookinetes while the postfertilization proteins, Pfs25 and Pfs28 help with the survival of ookinetes.^10-12^ In a phase-1 dose-escalation study in healthy adults involving the use of plant-produced Pfs25 virus-like particle as a transmission-blocking vaccine for malaria,^13^ it is reported that the vaccine had an acceptable safety and tolerability profile but had a weak transmission-reducing activity. According to Scaria et al,^14^ the preclinical and clinical investigations with protein-protein conjugates of Pfs25 and Pfs230 domain 1 demonstrated the feasibility of TBV. Fusing 2 or more antigens to form a chimeric protein has been reported to improve antigen production, stimulate a better immune response, inhibit parasite growth, and increase functional antibody levels.^15-17^ The efficacy of fused antigens can be assessed by their ability to elicit an immune response in the host.

Although the new chimeric epitopes may not have a 100% match with proteins in the pathogen, experimental results of 2 fused antigens have demonstrated promising results as vaccine candidates. According to Wu et al,^18^ there was strong transmission-blocking activity in rats with a recombinant Pfs48/45 fused to maltose-binding protein. Combining monoclonal antibodies of Pfs230D1 and Pfs48/45 resulted in a significant increase in transmission-reducing activity compared with individual monoclonal antibodies.^19^ The chimeric proteins that resulted from the fusion of Pfs230 and Pfs48/45 proteins were expressed in *Lactococcus lactis*.^20^ An important highlight from the study was that one of the chimeric proteins elicited more than threefold higher transmission-blocking antibody response in comparison with a single antigen alone. It was reported that the chimeric proteins induced antibodies with more than 80% reducing assay and therefore concluded that those chimeric proteins were potential next-generation vaccine candidates. The fusion of *Plasmodium berghei* 22 (Pb22) and *Plasmodium berghei* 37 (Pbg) induced robust and significantly higher levels of transmission-blocking activity relative to the administration of antigens individually in dual-antigen-immunized mice.^21^

Poor immunogenicity was reported for some vaccines designed from the combination of 2 antigens such as the combination of Pfs25 & Pfs28 and Pfs25 & Pfs230^22^ while others such as the combination of Pfs230 and Pfs48/45 elicited strong transmission-blocking antibodies responses against *P. falciparum*.^20^ However, a recent study reported the combination of mRNA of Pfs25 and Pfs230D1 to yield strong immune responses in mice.^14^ To block the transmission of the malaria parasite from mosquitoes to humans, particularly in endemic local settings, this work used not just the proteins or the fragments of the proteins of the TBV antigens, but highly antigenic peptides screened out of 4 TBV antigens to computationally design vaccine candidate capable of inducing an effective immune response against *P. falciparum*. The objectives were to (1) use computational tools to identify and select potential antigenic peptides expressed by both pre- and postfertilization gametocytes, ensuring both are included in the vaccine construct, (2) evaluate the ability of the selected peptides for cross-protection across multiple species of *Plasmodium*. (3) construct fused antigens from the selected antigenic peptides and optimize with suitable adjuvants, (4) assess whether the fused antigen vaccine candidate (FAVC) can still provide cross protections across the species of *Plasmodium*, (5) predict and evaluate the structural properties of the constructed vaccine candidates, and (6) use *in silico* analysis to evaluate the immunogenic properties of the fused antigens. Information on this type of vaccine design is limited in the literature. It is hoped that the fused antigens as candidate vaccines after *in vitro* and *in vivo* validation will elicit a better immune response against *P. falciparum*.

## Materials and Methods

### Sequence retrieval, alignment, and prediction of antigenic peptides

Complete protein sequences of *P. falciparum* antigens including Pfs25, Pfs28, Pfs48/45, and Pfs230 were downloaded from the National Center for Biotechnology Information (NCBI) (https://www.ncbi.nlm.nih.gov/protein) in a fasta format. Pfs230 and Pfs48/45 were prefertilization while Pfs25 and Pfs28 were postfertilization gametocytes of *P. falciparum*. The downloaded protein sequences underwent a blast search against their respective reference sequences which were XP_001347587.1 (Pfs25), XP_001347586.1 (Pfs28), XP_001350181.1 (Pfs48/45), and XP_001349600.1 (Pfs230). From PlasmoDB (https://plasmodb.org/plasmo/app/), the reference protein sequences mentioned belong to *Plasmodium falciparum* aligned with the gene sequence (PF3D7_1031000) encoding Pfs25, (PF3D7_1030900) encoding Pfs28, (PF3D7_1346700) encoding Pfs48/45 and (PF3D7_0209000) encoding Pfs230. The sequences that resulted from the blast were submitted to ClustalW (https://www.genome.jp/tools-bin/clustalw) with default parameters to perform multiple sequence alignments and only peptides with 15 or more amino acids were selected. The antigenicity test was carried out by submitting the aligned sequence to VaxiJen 2.0 (http://www.ddg-pharmfac.net/vaxijen/VaxiJen/VaxiJen.html)^23^ with a threshold of 0.5 and the parasite was selected as the target organism. Unique peptides were used for further analysis. The unique antigenic peptides were submitted to TMHMM 2.0 (https://services.healthtech.dtu.dk/service.php?TMHMM-2.0)^24^ for topology analysis. The peptides with outer membrane were then selected for additional analysis. The outer membrane peptides were submitted to AllerTOP (https://www.ddg-pharmfac.net/AllerTOP/)^25^ for an allergenicity test. Based on the allergenicity score, the top peptides that were both outside membrane and nonallergens were submitted for blast searching against *Homo sapiens* using NCBI blastp to exclude peptides that have similarities with human proteome. Also, the resulting peptides were searched against other species of *Plasmodium* to evaluate cross-protection of the predicted peptides for vaccine design.

### Construction of FAVC

Fused antigen has the potential to increase antibody responses toward vaccine effectiveness. An FAVC was constructed by selecting the peptides with the highest antigenicity scores with outside membrane, nonallergens, and without similarity with human proteome. This was done for each of the antigens at pre- and postfertilization gametocytes of *P falciparum*. To increase immunogenicity as well as the shelf life of the vaccine, an adjuvant is added to the vaccine formulations. In this study, 2 adjuvants, namely, Flagellin *Salmonella enterica* (FSE) and Cholera toxin B (CTB) were added as adjuvants to the N-terminal of the construct while “EAAAK” was used to link both the adjuvants and the antigens together. Linkers are used to alter junctional immunogenicity. EAAAK was chosen as the linker due to its unique structural and functional properties suitable for a fusion of proteins such as an α-helix-forming linker, enhanced solubility due to the presence of charged residues such as lysine, and nonimmunogenic (it means, it will not generate an unwanted immune response that can be recognized as a foreign substance by the immune systems)^26,27^ EAAAK as a linker has been extensively studied to be used in the fusion of proteins.^26,27^ Two vaccine candidates were formulated based on the adjuvants to give FAVC-FSE and FAVC-CTB. The FAVCs were further analyzed for antigenicity and allergenicity.

### Physicochemical, structural, immunoinformatic, and immunological studies of the FAVCs

#### Physicochemical and structural analyses of the FAVC

Determination of the physicochemical properties of FAVC was carried out using Protparam (https://web.expasy.org/protparam/).^28^The physicochemical parameters estimated included molecular weight, amino acid composition, isoelectric point, instability index, aliphatic index, the grand average of hydropathicity (GRAVY), the total number of negatively and positively charged residues, and the estimated half-life. Determining the structure of the fused antigen is important to understand the level of folding stability and amino acid interactions. The FAVC was analyzed for secondary structure with SOPMA (https://npsa-prabi.ibcp.fr/cgi-bin/npsa_automat.pl?page=/NPSA/npsa_sopma.html).^29^ The 3D structure of FAVC was generated with Robetta (https://robetta.bakerlab.org/submit.php), iDrug (https://drug.ai.tencent.com/console/en/tfold?type=predict), and trRosetta (https://yanglab.qd.sdu.edu.cn/trRosetta/)^30^ and thereafter was refined with GalaxyWeb (https://galaxy.seoklab.org/cgi-bin/submit.cgi?type=REFINE).^31^ Validation of FAVC was carried out with proSA-web (https://prosa.services.came.sbg.ac.at/prosa.php)^32^ to generate Z-score, ERRAT that gave overall quality and PROCHECK (https://saves.mbi.ucla.edu/)^33,34^ and SWISS-MODEL (https://swissmodel.expasy.org/assess)^35^ to generate Ramachandran plot. Thermodynamic properties with Scoop v1.0 (http://babylone.3bio.ulb.ac.be/SCooP/k_query.php)^36^ were used to estimate the thermodynamic properties of FAVC.

#### Prediction of antigenic epitopes from the selected antigenic peptides and adjuvants

The IEDB online resources (http://tools.iedb.org/mhci/) and (http://tools.iedb.org/mhcii/) were used for the prediction of cytotoxic (CD8^+^) (9 amino acids) and helper (CD4^+^) T-lymphocyte epitopes (15 amino acids) respectively for their human leukocyte antigen (HLA) allele binding. The generated epitopes with IC_50_ ⩽ 250 were selected along with the antigenicity score ⩾0.5 for CD8^+^ and CD4^+^. Conformational B-cells of the FAVC were predicted from the refined 3D structure with the ElliPro server (http://tools.iedb.org/ellipro/) ^37^ while the linear B-cells were predicted (threshold of 0.51 and window length of 16) using the ABCpred online server (https://webs.iiitd.edu.in/raghava/abcpred/ABC_submission.html).

#### Population coverage

Population coverage was analyzed to evaluate the effectiveness of the selected peptides against the population of the world. The online IEDB population coverage analysis tool (http://tools.iedb.org/population/) was used with epitope specificity of known HLA restrictions. The predicted HLA alleles for each epitope were used as input data.

#### In silico immune response simulation

The immune response was simulated using C-IMMSIM online tools (https://150.146.2.1/C-IMMSIM/index.php?page=1)^38^ to characterize the immunogenicity of the vaccine constructs. The C-IMMSIM tool is known to simulate the immunological response by combining the sequences of antigenic epitopes with the lymphocyte receptors using position-specific sore matrix and machine learning.^38^ All the parameters were default settings with an injection of vaccine (no LPS) except that the 3 injections were carried out with simulation steps of 1000 and the time steps for injection were 1, 85, and 170.

#### Disulfide linkage

Disulfide bond engineering was introduced to improve the stability of the constructed FAVC. The refined 3D structures of the FAVCs were first prepared using the protein preparation wizard in Maestro 12.8 of the Schrodinger 2021 version. Thereafter, the prepared structures were subjected to cysteine scanning using the cysteine mutation panel to select potential residue pairs that could be mutated to cysteine. The following criteria were used; Cys-Cys and Cys-X were excluded during filtering, minimum sequence separation was within 2.0 Å, C_β_—-C_β_ distance within 5.0 Å, solvent accessible surface area kept below 80 Å,^2^ and refinement with implicit solvent minimization.

### Molecular docking and molecular dynamics simulation of the vaccine construct

Bacterial flagellin used mostly in the development of chimeric malaria vaccine is recognized by Toll-like receptor 5 (TLR5).^39,40^ The structure for TLR5 (3v47) was downloaded from RCSB PDB^41^ and chain A was selected. The cleaning and preparation of the structure was done with UCSF Chimera v.1.14.^42^ The binding sites for both TLR5 and FAVC were determined for solvent accessibility and flexibility using Naccess 2.1.1 package.^43^ The TLR5 and FAVC were submitted to the HDOCK server (http://hdock.phys.hust.edu.cn/) for molecular docking and the residues for binding sites previously determined were specified.^44^ The binding interactions between TLR5 and FAVC were generated with LigPlot+ version 2.2.8.^45^ The best model with the lowest energy value was selected and visualized with UCSF Chimera.

Following molecular docking, the best-docked complex and FAVC were subjected to molecular dynamics (MD) simulation to assess the level of stability and interaction between TLR5 and FAVC using AMBER 14.^46^ The proteins were described using FF14SB^47^ with the LEAP module to generate the topologies of the proteins by adding protons and Na^+^ as counter ions. The systems were solvated in an orthorhombic solvation box of TIP3P 8 Å water model.^48^ To achieve the lowest energy for the protein, initial energy minimization was carried out.^49^ The minimization process was executed with 10 000 steps at first (500 steepest descents with 9500 conjugate gradient) and this was followed by full minimization of 2000 steps. The system was heated up in a canonical ensemble (NVT) for 2 ns with a Langevin thermostat ranging between 0 and 300 K using a collision frequency of 1.0 ps^-1^. The water system density was regulated at 4 ns of NPT simulation. The system equilibrium was attained at 300 K for an additional 2 ns at the pressure of 1 bar. The MD production was run for 300 ns of NPT using AMBER 18 package.^50^ The molecular mechanics and generalized born surface area (MM/GBSA) module in AMBER 14 was used to determine the endpoint binding free energy of the docked complex. Following the molecular dynamics simulation, the PTRAJ and CPPTRAJ modules in AMBER 14 were employed to generate 2000 ensemble structures of the complexes, root mean square deviation (RMSD), root mean square fluctuations (RMSF), solvent accessible surface area (SASA), and number of hydrogen bonds (NHB). The generated structure was viewed with VMD v 1.9.3.^51^ The normal mode analysis (NMA) using MDM-TAST-web server^52^ was performed on 10 frames of the complexes. Principal component and cross-correlation analyses were performed on 2000 ensemble structures with Bio3D package in RStudio v 4.0.4.^53,54^

## Results

### Generation of antigenic peptide for vaccine construction

A total of 175, 5, 389, and 304 sequences for Pfs25, Pfs28, Pfs48/45, and Pfs230 were retrieved, respectively, from NCBI. The blast results produced 88 (Pfs25), 2 (Pfs28), 85 (Pfs48/45), and 94 (Pfs230) sequences (Supplemental Table S1). Following the alignment of sequences from the blast, the unique peptides generated were 11 for Pfs25, 3 for Pfs28, 39 for Pfs48/45, and 71 for Pfs230. The numbers of peptides that met the condition of antigenicity of ⩾0.5 thresholds were 11 (Pfs25), 3 (Pfs28), 34 (Pfs48/45), and 36 (Pfs230) (Supplemental Tables S2 to S5). There were 8, 3, 28, and 31 peptides located outside the membrane for topology analysis for Pfs25, Pfs28, Pfs48/45, and Pfs230 in the order listed (Supplemental Tables S6 to S9).

In descending order of antigenicity score, among the best 10 peptides that were outside the membrane and were nonallergens; 2, 2, 5, and 5 were selected for Pfs25, Pfs28, Pfs48/45, and Pfs230, respectively (Table 1). Some of the peptides presented in Table 1 may look identical and therefore, the difference in the peptides is colored as presented in Supplemental Table S9A. These differences are also reflected in their antigenic scores presented in Table 1. A single change in amino acid sequences could have significant effects on the protein properties.^55,56^ Therefore, a single change in the amino acid of the peptides presented was not neglected in the present study. To evaluate whether the predicted peptides could elicit cross-protection, the sequences were submitted for similarity search across the *Plasmodium* species. The result is presented in Supplemental Table S9B with query cover, E-value, percentage identity and protein accession number. The E-value ranged between 9.00 ×10^-24^ and 6.00 ×10^-5^ for *P malariae*, 8.00 ×10^-18^ and 2.00 ×10^-7^ for *P knowlesi*, 3.00 ×10^-19^ and 4.00 ×10^-5^ for *P ovale*, and 1.00 ×10^-17^ and 3.00 ×10^-6^ for *P vivax*. The percentage identity ranged between 42.42 and 70.49 for *P malariae*, 46.15 and 62.26 for *P knowlesi*, 32.20 and 58.49 for *P ovale*, and 44.74 and 65.38 for *P vivax*.

**Table 1.** The final selected peptides (with highest antigenicity scores, outside membrane, nonallergens, and without similarity with human proteome) for vaccine construction.

Antigen		Peptide	Number of residues	Antigenicity	Protein accession number	Position of the peptide
Pfs25	P1	ILDTSNPVKTAVCSCNIGKVPNVQNQNKCSKDGETKCSLKCLKENETCKAVDGIYKCDCK	60	0.7589	UIT08906.1	120-180
	P2	DGFIIDNESSICTAFSAYNILNLSIMFILFSVCFFIM	37	0.5775	AAD39544.1	180-217
Pfs28	P3	MNTYFKVLLFLFIQLYITLNKARVTENTICKYGYLIQMSNHYECKCIEGYVLINEDTCGK	60	0.9098	AAT00624.1	1-60
	P4	SNGGGNTVDQADTSYSVINGVTLTHVLIVCSIFIKLLI	38	0.5793	AAT00624.1	180-218
Pfs48/45	P5	SSNDSSKHTFTDSLDISLVDDNAHISCNVHLSEPKYNHLVGLNCPGDIVPDCFFLVYQPE	60	1.0393	AAL74377.1	300-360
	P6	SSNDSSKHTFTDSLDISLVDDNAHISCNVHLSEPKYNHLVGLNCPGDIIPDCFFQVYQPE	60	1.0172	ABO41490.1	293-353
	P7	MMLYISAKKAQVAFILYIVLVLRIISGNNDFYKPSSLNSEISGFIGYKCNFSNEGVHNLK	60	0.6462	AAL74379.1	1-60
	P8	MMLYISAKKAQVAFILYIVLVLRIISGNNDFYNPSALNSEISGFIGYKCNFSNEGVHNLK	60	0.6224	AAL74380.1	1-60
	P9	MMLYISAKKAQVAFILYIVLVLRIISGNNDFCKPSSLNSEISGFIGYKCNFSNEGVHDLK	60	0.6155	AAL74350.1	1-60
Pfs230	P10	TKLKENLLSKLIYGLLISPTVNEKENNFKE	30	1.1821	UIT08906.1	124-154
	P11	EEEEYDDYVYEESGDETEEQLQEEHQEEVGAESSEESFNDEDEDSVEARDGDMIR	55	0.9218	AAA29734.1	300-355
	P12	VDTGPVLDNSTFEKYFKNIKIKPDKFFEKVINENDDTEEEKDLESILPGAIVSPMKVLKK	60	0.9154	AAG12332.1	1800-1860
	P13	VEEGVQNEEYKKFSLKPSLVFDDNNNDIKVIGKEKNEVSISLALKGVYGNRIFTFDKNGK	60	0.9056	AAA29734.1	2000-2060
	P14	EEEEEYDDYVYEESGDETEEQLQEEHQEEVGAESSEESFNDEDEDSVEARDGDMIRVDEY	60	0.8761	AAG12332.1	300-360

### Physicochemical and structural properties of fused antigen candidate vaccine

For easy synthesis and purification, only the first best peptide from each antigen (P1, P3, P5, and P10) from Table 1 was selected for the construction of FAVC. Two vaccine candidates were constructed with 2 different adjuvants, namely, FSE and CTB to form FAVC-FSE and FAVC-CTB, respectively (Supplemental Figure S1). To assess whether the FAVCs can still provide cross protections across the species of *Plasmodium*, the sequences were submitted for similarity search across the *Plasmodium* species. According to the results provided in Supplemental Table S9C, the range of E-value is 2.00 ×10^-13^ and 3.00 ×10^-12^ (*P vivax*), 3.00 ×10^-15^ and 3.00 ×10^-13^ (*P ovale*), 4.00 ×10^-19^ and 1.00 ×10^-3^ (*P malariae*), and 5.00 ×10^-16^ and 2.00 ×10^-9^ (*P knowlesi*) for FAVC-FSE and 3.00 ×10^-14^ and 2.00 ×10^-12^ (*P vivax*), 8.00 ×10^-16^ and 1.00 ×10^-13^ (*P ovale*), 3.00 ×10^-21^ and 4.00 ×10^-4^ (*P malariae*), and 2.00 ×10^-16^ and 6.00 ×10^-10^ (*P knowlesi*) for FAVC-CTB. The E-values reported cut across the strains of the mentioned species of *Plasmodium*. The E-values observed for the fused antigens were much better than the ones observed for the peptides in section 3.1.

**Figure 1.**
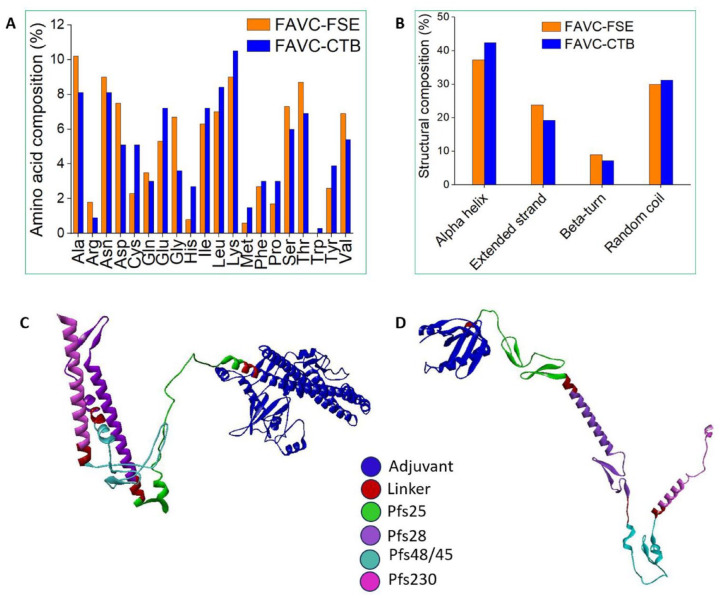
(A) The amino acid composition (%) of FAVCs, (B) Secondary structure composition of FAVCs and the refined 3D structures of (C) The FAVC-FSE and (D) The FAVC-CTB, (C).

**Figure 2.**
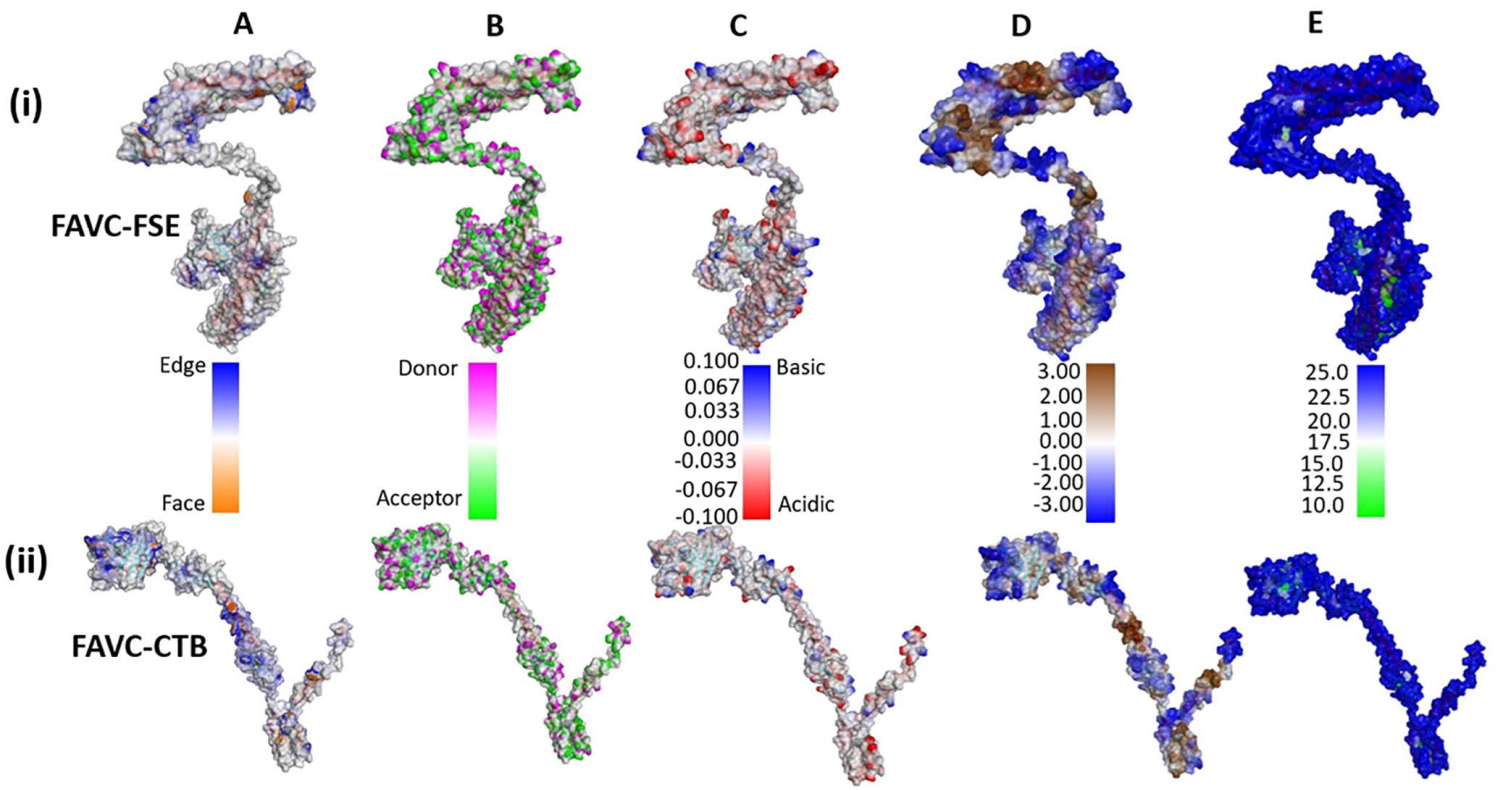
Detailed qualities of amino acid residues with (i) for FAVC-FSE and (ii) for FAVC-CTB. The keys describing the colors of the structures are placed in between them. (A) Aromatic residues (B) hydrogen bonds (C) interpolated charge distribution (blue, white, and red represent positive, neutral, and negative charged residues, respectively), (D) red to blue representing hydrophobicity to hydrophilicity, respectively, and (E) solvent accessibility surface (blue for exposed and green for buried residues).

The amino acid composition of the FAVCs is displayed in Figure 1A while all other physicochemical properties of the FAVCs as analyzed by the ProtParam tool are displayed in Table 2. The amino acid composition presented in Figure 1A showed that alanine had the highest value of 10.2% for FAVC-FSE and lysine of 10.5% for FAVC-CTB. The N-terminal of both FAVCs was Glycine with a half-life estimated as 30 and 7.2 hours for FAVC-FSE and FAVC-CTB, respectively in mammalian reticulocytes, 20 hours for yeast, and 10 hours in *Escherichia coli* all in vivo. In Table 2, the instability indexes computed were less than 40 which classified the proteins as stable. The grand average of hydropathicity (GRAVY) showed that the FAVCs were hydrophilic having negative values. The aliphatic indices of 81.95 and 84.68 are indications of an increase in their thermal stability. For comparison of models, the physicochemical properties of the FAVCs that are available with other tools are presented in Supplemental Table S10. The tools are ThermoFisher peptide analyzing tool (https://www.thermofisher.com/za/en/home/life-science/protein-biology/peptides-proteins/custom-peptide-synthesis-services/peptide-analyzing-tool.html), Prot pi protein/peptide calculator (https://www.protpi.ch/Calculator/ProteinTool#Results), and Biosynth peptide calculator (https://www.biosynth.com/peptide-calculator). The GRAVY calculated were the same for all the tools (as also presented in Table 2), the PI ranged between 5.0-5.21 (FAVC-FSE) and 6.30-6.37 (FAVC-CTB), while net charge at pH 7.4 ranged between -14.43 to -15.19 (FAVC-FSE) and -4.48 to -5.42 (FAVC-CTB). The results of the secondary structure (analyzed by SOPMA) of FAVC-FSE and FAVC-CTB are presented in Figure 1B with the highest percentage recorded for alpha helix (37.25% and 42.34% FAVC-FSE and FAVC-CTB, respectively). From Supplemental Table S11, the predicted helix composition with the other models (GOR4, https://npsa-prabi.ibcp.fr/cgi-bin/npsa_automat.pl?page=/NPSA/npsa_gor4.html, and PROTEUS2, http://www.proteus2.ca/proteus2/) ranged between 28.00% and 37.25% for FAVC-FSE and 23.00% and 47.34% for FAVC-CTB, extended strand ranged between 20.61% and 23.82% for FAVC-FSE and 19.22% and 21.02% for FAVC-CTB, beta turn/sheet ranged between 9.00% and 23.00% for FAVC-FSE and 7.21% and 32.00% for FAVC-CTB and random coil ranged between 29.92% and 50.00% for FAVC-FSE and 31.23% and 51.05% for FAVC-CTB. The 3D structures of FAVCs were determined with Robetta, trRosetta and iDrug as presented in Supplemental Table S12 with their Ramachandra plot analysis. The best quality was selected with trRosetta and refined with Galaxy as presented in Figure 1C and D. Observing Figure 1C and D, the structures are not the same despite only the adjuvants differentiated them. This is because the incorporation of different adjuvants into the peptides to form chimeric vaccine constructs can impact the overall 3D structures of the resulting peptides.^57^ This is primarily due to the interactions of the adjuvants with the peptides that can lead to folding, stability and conformational dynamics of the peptide. This may be influenced by factors such as steric hindrance (bulky adjuvants), hydrogen bond formation and electrostatic attractions.^57^ The refined 3D structures were analyzed for their qualities based on Z-score, Ramachandra, and Errat with the results presented in Table 2. Further structural qualities of the FAVCs based on the residual contents are presented in Figure 2. This analysis is essential to gain more insights into the amino acid compositions that can affect protein folding, stability, function and interaction.^58^ Hydrophobic residues stabilize the proteins by clustering in the core of the proteins thereby shielding the proteins from an aqueous environment and charged residues through ionic interaction and hydrogen bonding contribute to the stabilization and solubility of the proteins.

**Table 2.** Physical and structural properties of fused antigen vaccine candidate (FAVC).

Property	FAVC-FSE	FAVC-CTB
Amino acid length	655.00	333.00
Molecular weight (kDa)	70.06	37.12
Number of atoms	9798.00	5194.00
Instability index	17.42	33.44
Aliphatic index	81.95	84.68
Grand average of hydropathicity	–0.39	–0.31
Total number of negatively charged residues (Asp + Glu)	84.00	41.00
Total number of positively charged residues (Arg + Lys)	71.00	38.00
Isoelectric point (PI)	5.19	6.31
Z score	–6.70	–4.79
Ramachandran favored regions (%) (SWISS)	96.94	94.86
Ramachandran favored regions (%) (PROCHECK)	93.20	90.60
Errat overall quality (%)	94.20	89.85

### Qualitative analysis of the vaccine constructs

#### Allergenicity and antigenicity

The FAVC-FSE and FAVC-CTB were nonallergen with an antigenicity score of 0.7135 and 0.7152, respectively.

#### The prediction of CD8^+^ and CD4^+^ T-cell epitopes from the vaccine constructs

The predicted CD8^+^ and CD4^+^ T-cell epitopes from P1, P3, P5, P10, FSE, and CTB are presented in Tables 3 and 4. The epitopes with antigenicity score cut-off of ⩾0.5 were recorded. The detailed information including the HLA alleles of MHC-I and MHC-II are presented in Supplemental Tables S13 and S14 for CD8^+^ and CD4^+^, respectively.

**Table 3.** The predicted CD8^+^ T-cell epitopes from P1, P3, P5, P10 and the adjuvants.

	CD8^+^	Antigenicity score		CD8^+^	Antigenicity score
P1	SKDGETKCS	2.7283	P10	SKLIYGLLI	1.9106
	CSKDGETKC	2.4277		LIYGLLISP	1.6789
	KCSKDGETK	2.409		TKLKENLLS	1.4642
	QNKCSKDGE	1.9355		KLIYGLLIS	1.428
	NQNKCSKDG	1.928		NEKENNFKE	1.363
	KDGETKCSL	1.9027		LSKLIYGLL	1.2205
	GETKCSLKC	1.8916		NLLSKLIYG	1.2149
P3	YFKVLLFLF	2.6377	FSE adjuvant	TKTGDDGNG	2.807
	FKVLLFLFI	2.5524		IKGGKEGDT	2.7496
	LFLFIQLYI	2.5186		DTKTGDDGN	2.694
	NTYFKVLLF	2.3689		AIKGGKEGD	2.5329
	TYFKVLLFL	2.1777		TNGTNSDSD	2.3579
	LFIQLYITL	1.9928		GDDGNGKVS	2.3053
	LLFLFIQLY	1.9535		DVKSLGLDG	2.2955
P5	GDIVPDCFF	2.8623	CTB adjuvant	TFQVEVPGS	2.3897
	PGDIVPDCF	2.7054		FQVEVPGSQ	2.2985
	NAHISCNVH	2.2105		ATFQVEVPG	2.2938
	CPGDIVPDC	2.0034		GATFQVEVP	1.4356
	DIVPDCFFL	1.9996		QVEVPGSQH	1.3731
	DDNAHISCN	1.8599		LNDKIFSYT	1.3338
	AHISCNVHL	1.8435		ERMKDTLRI	1.2089

**Table 4.** The predicted CD4^+^ T-cell epitopes from P1, P3, P5, P10 and the adjuvants.

	CD4^+^	Antigenicity score		CD4^+^	Antigenicity score
P1	DTSNPVKTAVCSCNI	0.8015	P10	LKENLLSKLIYGLLI	1.6029
	ENETCKAVDGIYKCD	0.7392		KENLLSKLIYGLLIS	1.4532
	KENETCKAVDGIYKC	0.6237		ENLLSKLIYGLLISP	1.3341
	TSNPVKTAVCSCNIG	0.6057		KLKENLLSKLIYGLL	1.273
	ETCKAVDGIYKCDCK	0.5663		LLSKLIYGLLISPTV	1.1842
P3	TYFKVLLFLFIQLYI	2.1585		TKLKENLLSKLIYGL	1.1451
	YFKVLLFLFIQLYIT	2.1055		LSKLIYGLLISPTVN	1.1016
	FKVLLFLFIQLYITL	2.0201	FSE adjuvant	GDDGNGKVSTTINGE	2.4132
	NTYFKVLLFLFIQLY	1.9872		TGDDGNGKVSTTING	2.0716
	FKVLLFLFIQLYITL	1.9736		KAIAGAIKGGKEGDT	1.9219
	MNTYFKVLLFLFIQL	1.817		AKAIAGAIKGGKEGD	1.8771
P5	GLNCPGDIVPDCFFL	2.0727		TKTGDDGNGKVSTTI	1.8599
	VGLNCPGDIVPDCFF	2.0698		NGKVSTTINGEKVTL	1.6593
	LNCPGDIVPDCFFLV	1.8761		KTGDDGNGKVSTTIN	1.6305
	LVGLNCPGDIVPDCF	1.8341	CTB adjuvant	GATFQVEVPGSQHID	1.1206
	NCPGDIVPDCFFLVY	1.8334		ERMKDTLRIAYLTEA	0.9987
	PGDIVPDCFFLVYQP	1.7606		FKNGATFQVEVPGSQ	0.8779
	NCPGDIVPDCFFLVY	1.7287		LNDKIFSYTESLAGK	0.8615
				TFKNGATFQVEVPGS	0.7831
				VEVPGSQHIDSQKKA	0.7431
				KDTLRIAYLTEAKVE	0.7325

#### Discontinuous and linear B-cell epitope prediction of fused antigen vaccine candidates

**Figure 3.**
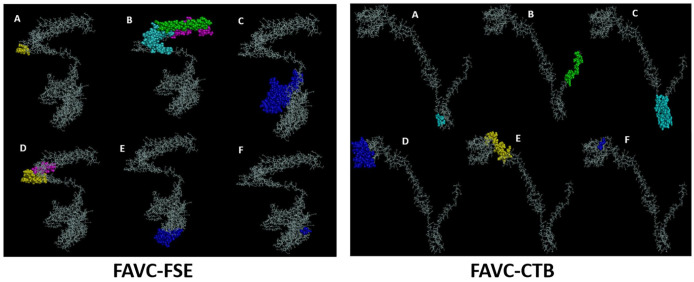
The 3D structure of the 6 predicted discontinuous B-cell epitopes (A-F) from the refined fused antigen vaccine constructs (FAVC-FSE and FAVC-CTB). The section in different colors (P1 = yellow, P3 = pink, P5 = cyan, P10 = green and adjuvant = blue) is the conformational B-cell epitope and the gray section is the remaining part of the residue.

With the 3D model of the refined FAVCs, the ElliPro server predicted 6 conformational B-cell epitopes (Figure 3). For FAVC-FSE, a total of 319 residues were generated from the conformational B-cell epitopes with scores between 0.513 and 0.903. The breakdown of the number of residues along with their scores were 8 (0.903), 143 (0.831), 82 (0.702), 31 (0.697), 51 (0.599), and 4 (0.513) for residues A, B, C, D, E, and F, respectively. For FAVC-CTB, a total of 176 residues were generated from the conformational B-cell epitopes with scores between 0.521 and 0.902. The breakdown of the number of residues along with their scores were 6 (0.902), 23 (0.83), 56 (0.755), 51 (0.687), 36 (0.617), and 4 (0.521) for residues A, B, C, D, E, and F, respectively. The linear B-cell epitopes for the antigenic peptides and the adjuvants are presented in Table 5 with antigenicity score ⩾ 0.5.

**Table 5.** Linear B-cell epitopes from P1, P3, P5, P10 and the adjuvants.

	B-cell	Antigenicity score
P1	NKCSKDGETKCSLKCL	1.6179
	VPNVQNQNKCSKDGET	1.0459
	TKCSLKCLKENETCKA	0.7017
	NETCKAVDGIYKCDCK	0.5711
P3	GYLIQMSNHYECKCIE	0.9325
	CKCIEGYVLINEDTCG	0.8001
	IQLYITLNKARVTENT	0.7038
	ENTICKYGYLIQMSNH	0.5333
P5	PGDIVPDCFFLVYQPE	1.6472
	LVDDNAHISCNVHLSE	1.3769
	YNHLVGLNCPGDIVPD	1.0713
	HLSEPKYNHLVGLNCP	0.6377
	TDSLDISLVDDNAHIS	0.5558
P10	YGLLISPTVNEKENNF	0.7491
FSE adjuvant	TGDDGNGKVSTTINGE	2.2212
	GKVSTTINGEKVTLTV	1.5059
	TFTIDTKTGDDGNGKV	1.4225
	DVKSLGLDGFNVNGPK	1.3773
	QATNGTNSDSDLKSIQ	1.3297
	AGAIKGGKEGDTFDYK	1.2325
	AVKGESKITVNGAEYT	1.2141
CTB adjuvant	QVEVPGSQHIDSQKKA	0.8855
	YTESLAGKDEMAIITF	0.7121
	NDKIFSYTESLAGKDE	0.6137
	SQHIDSQKKAIERMKD	0.577
	AIERMKDTLRIAYLTE	0.5196

#### Population coverage analysis

Population coverage of combined MHC-I and MHC-II were studied based on their respective HLA alleles, and the results are presented in Table 6 for different geographical areas of the world. The detailed analysis for different countries is presented in Supplemental Table S15 with a cut-off of ⩾70%. In Table 6, the world population coverage is 94.41%. Among the regions of the world, the highest coverage was recorded for East Asia (96.94%). According to Supplemental Table S15 detailed for the countries of the world, Ireland Northern has the highest population coverage of 98.69%. The high population coverage recorded for the selected epitopes from the antigenic peptides suggested the effectiveness in tackling malaria infection globally.

**Table 6.** Population coverage (%) for world and geographic regions of combined MHC-I and MHC-II selected epitopes.

Area	Coverage
East Asia	96.94
Europe	96.11
North America	94.86
World	94.41
Oceania	89.98
North Africa	89.18
South Asia	89.16
Southeast Asia	88.97
Northeast Asia	88.43
West Africa	86.37
South Africa	84.54
Central Africa	84.23
West Indies	83.79
South America	81.83
East Africa	78.47
Southwest Asia	75.13
Central America	25.97

#### Immune simulation of the vaccine constructs

**Figure 4.**
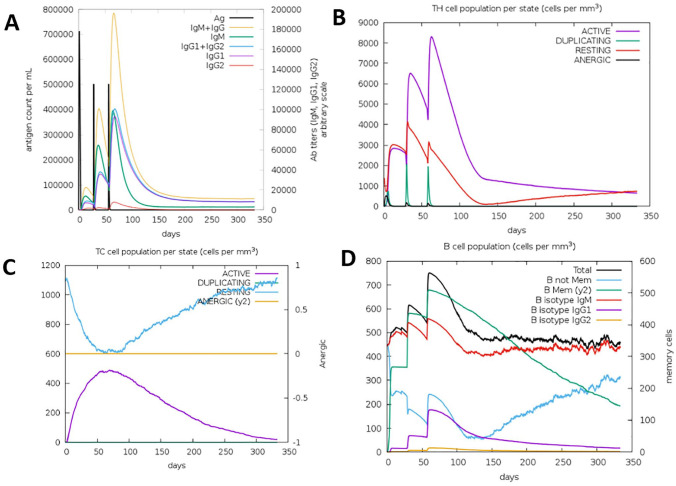
The plots relatives to the C-IMMSIM immune simulation of the predicted epitopes of FAVC-FSE (A) immunoglobulins and immunocomplexes in response to antigen injection, (B) T-helper lymphocytes count, (C) T-cytotoxic lymphocytes count, and (D) the induction of B lymphocytes populations after antigen injections.

**Figure 5.**
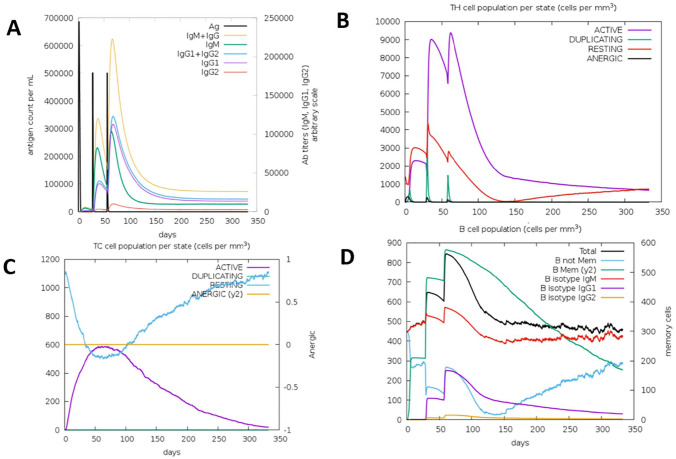
The plots relatives to the C-IMMSIM immune simulation of the predicted epitopes of FAVC-CTB (A) immunoglobulins and immunocomplexes in response to antigen injection, (B) T-helper lymphocytes count, (C) T-cytotoxic lymphocytes count, and (D) the induction of B lymphocytes populations after antigen injections.

The humoral and cellular response of the receptor against the vaccine constructs was estimated using the C-IMMSIM online tool after 3 doses of FAVCs had been administered. As observed from Figures 4A and 5A, the gradual increase in the level of immunoglobulin (IgG1, IgG2, IgM, IgG1 + IgG2, and IgM + IgG) after the first, second, and third injections indicated a significant simulation by the immune response against the FAVCs. The immune simulation of the FAVCs generated higher responses suggesting activation of the memory cells. This, in turn, leads to an increase in the peaks of the antibodies with high levels of immunoglobulin activity. There was an indication that T-cells increased in response to each antigen exposure because both TH (helper) and TC (cytotoxic) had a strong response in association with memory acquisition (Figure 4B and C, Figure 5B and C). From Figures 4D and 5D, a decrease in antigen concentration because of the increase in memory cells was observed for B-cell populations. The rest of the plots for the active B lymphocyte population, dendritic cells, macrophages and production of cytokines and interleukins are presented in Supplemental Figures S3 and S4.

### Disulfide linkage for FAVC stability

**Figure 6.**
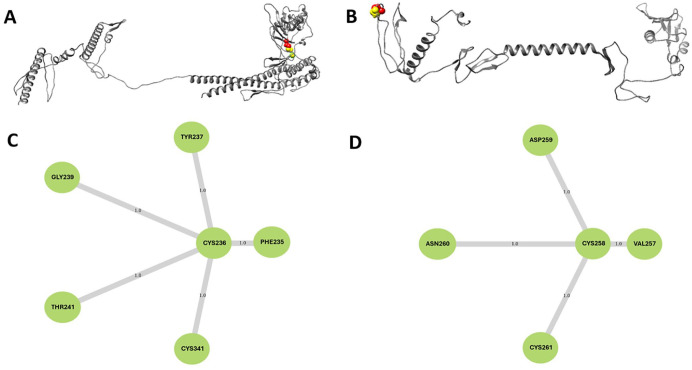
Disulfide engineering with residues undergoing disulfide bond in yellow and red (A) FAVC-FSE and (B) FAVC-CTB. A weighted residue contact map with a cut-off distance of 6.7 Å and step size of 1.0 for (C) FAVC-FSE and (D) FAVC-CTB. The selected residue is displayed at the center and surrounded by its neighboring residues.

A total of 55 and 46 pairs of potential residues were obtained for FAVC-FSE and FAVC-CTB, respectively, which could be used in disulfide engineering. From these numbers, only 1 residue pair was selected based on the criteria of the lower weighted score (between 200 and 500), change in interaction energy (ΔEi) score and change in strain energy (ΔSE) score. The ASP236 and ASP341 for FAVC-FSE and ASP258 and ALA 261 FAVC-CTB were mutated to cysteine residues. The CYS236/341 residue pairs had a weighted score of 426.30, ΔEi of -250.58 and ΔSE of -60.93 for FAVC-FSE while CYS258/261 with a weighted score of 411.66, ΔEi of -128.86 and ΔSE of 8.0 for FAVC-CTB. The mutant 3D structures following disulfide engineering for FAVC-FSE are presented in Figure 6A and B. A weighted residue contact map^52^ was also calculated for the mutant structure at residue 236 and 258 for FAVC-FSE and FAVC-CTB, respectively. This is to describe the contact frequencies of the selected residue and its neighbors with a cut-off distance of 6.7 Å and step size of 1.0. As can be seen from Figure 6C and D, PHE235, TYR237, GLY239, THR241, and CYS341 surrounded CYS236 as locust of interest residue for FAVC-FSE while VAL257, ASP259, ASN250, and CYS261 surrounded CYS258 as locust of interest residue for FAVC-CTB. There was no physical difference observed in the 3D structures before and after disulfide linkage as presented in Supplemental Figure S2. Ramachandran plot was used to check the quality of the mutant structures. When the PROCHECK tool was used, the results were not different from the ones presented in Table 2 (before and after disulfide bridging) but when SWISS-MODEL was used, the result showed Ramachandran favored at 96.94% (before) and 94.49% (after) for FAVC-FSE, and 94.86% (before) and 93.05% (after) for FAVC-CTB.

### Molecular docking and molecular dynamics simulation of FAVC with Toll-like receptor 5

The FAVCs were docked into the binding domain of TLR5 using the HDOCK online docking tool to predict the interaction between FAVC and TLR5. Supplemental Table S16 displayed the amino acid residues of TLR5 generated with the Naccess 2.1.1 package and these were used as the binding sites of TLR5 submitted for molecular docking of the FAVCs. Supplemental Figure S5(A) presented the binding sites of bacterial flagellin (ligand) mapped out on TLR5 (receptor). Comparing amino acid residues, almost all the binding sites of TLR5 in Supplemental Figure S5(A) were similar to those of Supplemental Table S16. After molecular docking, most of the residues displayed in Supplemental Table S16 were involved in the binding of the FAVCs as presented in Supplemental Figures S5(B) and (C) mapped for FAVC-FSE and FAVC-CTB, respectively.

**Figure 7.**
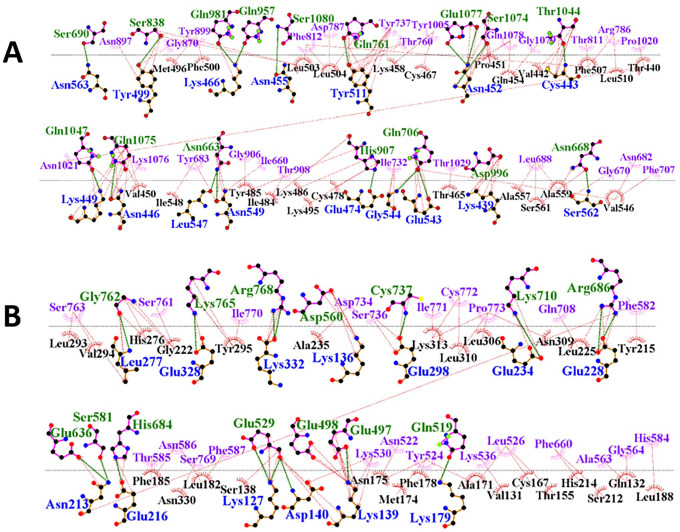
Receptor-fused antigen (TLR5-FAVC) interactions as analyzed by LigPlot+. (A) The TLR5-FAVC-FSE. (B) The TLR5-FAVC-CTB. The dotted green bonds were represented by hydrogen bond interaction while the dotted red bonds were represented by hydrophobic interaction. The TLR5 residues were printed in green while those of FAVC were printed in blue. The residues printed in purple and black were involved in hydrophobic interaction. TLR5 = Toll-like receptor 5; FAVC = Fused antigen vaccine candidate; FSE = Flagellin *Salmonella enterica*; CTB = Cholera toxin B.

**Figure 8.**
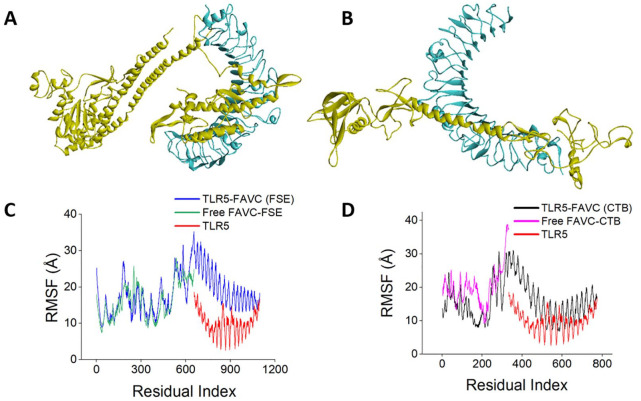
Molecular dynamics simulation at 300 ns simulation time. TLR5-FAVC complex (TLR5 in cyan, FAVC in yellow): (A) TLR5-FAVC (FSE). (B) TLR5-FAVC (CTB). Root Mean Square Fluctuations (RMSF): (C) TLR5-FAVC (FSE). The residual index for FAVC is 1 to 655 while TLR5 is 656 to 1096. (C) TLR5-FAVC (CTB). The residual index for FAVC is 1 to 333 while TLR5 is 334 to 773. TLR5 = Toll-like receptor 5; FAVC = Fused antigen vaccine candidate; FSE = Flagellin *Salmonella enterica*; CTB = Cholera toxin B.

**Figure 9.**
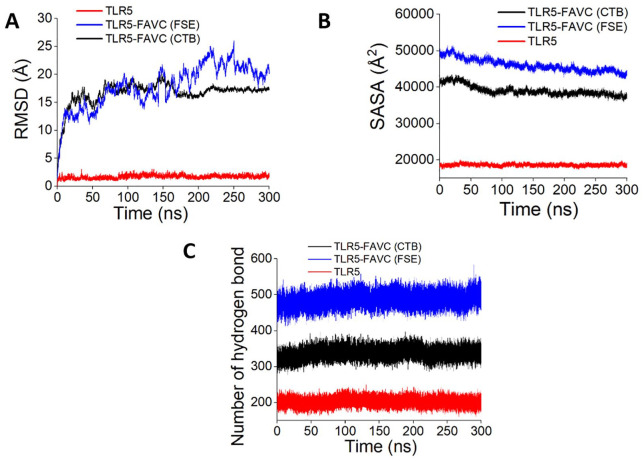
Molecular dynamics simulation at 300 ns simulation time. (A) Root Mean Square Deviation (RMSD). (B) Solvent Accessible Surface Area (SASA). D. Number of hydrogen bonds. TLR5 = Toll-like receptor 5; FAVC = Fused antigen vaccine candidate; FSE = Flagellin *Salmonella enterica*; CTB = Cholera toxin B.

The best-docked complexes generated from molecular docking were submitted for MD simulation to further study the interactions and stability of the TLR5-FAVC complex (Figures 7 and 8A and B). Some of these interactions are displayed in Figure 7 with 18 hydrogen bonds, 89 hydrophobic interactions and 1 salt bridge which existed between ASP 996 (TLR5) and LYS 439 (FAVC) for TLR5-FAVC-FSE (Figure 7A) and 16 hydrogen bonds, 73 hydrophobic interactions and 1 salt bridge which existed between GLU 498 (TLR5) and LYS 139 (FAVC) for TLR5-FAVC-CTB (Figure 7B). It should be noted that the numbering of amino acids of TLR5 is different after molecular dynamic simulations because the Amber force field used during the simulation renumbered the residues. The binding affinities of TLR5 with FAVC were calculated using MM/GBSA. The calculated energies presented in Table 7 represented the average values over 300 ns. The binding energies (∆G_bind_) for the complexes were -67.47 and -69.14 kcal/mol for TLR5-FAVC-FSE and TLR5-FAVC-CTB, respectively, with contributions from van der Waals, electrostatic, polar solvation, nonpolar solvation, gas phase, and solvation-free energy. As shown in Table 8 for TLR5-FAVC-FSE, the binding energy decomposition (with cut-off ⩽ -1.0) for the important residues ranged between -4.146 (PHE507) and -1.003 kcal/mol (GLN454) for FAVC while that of TLR5 ranged from -3.622 (TYR1005) to -1.030 kcal/mol (THR1029). In Table 9 for TLR5-FAVC-CTB, the binding energy decomposition (with cut-off ⩽ -1.0) for the important residues ranged between -5.260 (PHE185) and -1.075 kcal/mol (ASN329) for FAVC while that of TLR5 ranged from -4.352 (ARG686) to -1.045 kcal/mol (SER769). Observing Supplemental Table S17, the residues displayed in Table 8 are from peptides P1, P3 and P5 for FAVC-FSE while the residues displayed in Table 9 are from P3, and P10 for FAVC-CTB. The stability of the TLR5-FAVC complexes was assessed using RMSD, RMSF, SASA and NHB. The residual index on the RMSF plot of the C-alpha atoms calculated across all residues of the complex, free FAVCs and TLR5 were presented in Figure 8C and D. For FAVC-FSE, fluctuations (at residual index regions 163-251, 386-483, 349-420, and 534-621) were observed in the overall protein flexibility of FAVC in both free and bound states that ran from 1 to 1096 (1-655 for FAVC and 656-1096 for TLR5). For FAVC-CTB, fluctuations (at residual index regions 1-44, 110-204, and 222-245) were observed in the overall protein flexibility of FAVC in both free and bound states that ran from 1 to 773 (1-333 for FAVC and 334-773 for TLR5). The observed fluctuations were due to the participation and interactions of FAVC in a bound state with TLR5. These fluctuations were also observed between the residual index of the TLR5 region of the complexes and that of TLR5 only. The RMSD results presented in Figure 9A for FAVC-FSE showed the maximum fluctuation at 24.73 Å and showed better stability between 68 and 106 ns (17.06 Å). For FAVC-CTB (Figure 9A), the RMSD showed maximum fluctuation at 19.72 Å and very stable between 211 and 300 ns (17.00 Å). The overall stability observed from the RMSD result showed that TLR5-FAVC-CTB is more stable than TLR5-FAVC-FSE. The stability of FAVC was further confirmed with the result of SASA as shown in Figure 9B which was much more reduced in TLR5-FAVC-CTB. The low values for SASA observed for the TLR5-FAVC-CTB indicated that the surfaces of the many interacting residues are no longer accessible as observed from the stronger binding and decomposition energies presented in Tables 7 and 9. It is evident from the plot of Figure 9C that the NHB is higher in TLR5-FAVC-FSE than in TLR5-FAVC-CTB. This is supported by the NHB displayed in Figure 7 which showed higher NHB in TLR5-FAVC-FSE than TLR5-FAVC-CTB. Observing Figure 9, the TLR5 only without binding of the vaccine shows less fluctuation and is more stable than the complexes. However, this may be expected as the TLR5 is a crystal structure that has been validated experimentally.^59^

**Table 7.** Energy component based on molecular mechanics and generalized born surface area (MM/GBSA) for TLR5-FAVC complex.

Energy Component	Energy (kcal/mol)	
	TLR5-FAVC-FSE	TLR5-FAVC-CTB
∆E_VDW_	–199.72	–141.62
∆E_EEL_	–242.33	–569.07
∆EG_EGB_	401.71	661.77
∆E_ESURF_	–27.13	–20.22
∆G_gas_	–442.05	–710.69
∆G_solv_	374.58	641.55
∆G_bind_	–67.47	–69.14

∆E_VDW_ = van der Waals interaction energies, ∆E_ELE_ = electrostatic contribution, ∆EG_EGB_ = polar solvation contribution, ∆E_ESURF_ = Nonpolar solvation energy, ∆G_gas_ = gas phase energy, ∆G_solv_ = solvation-free energy, and ∆G_bind_ = binding free energy, TLR5 = Toll-like receptor 5, FAVC = Fused antigen vaccine candidate.

**Table 8.** Decomposed binding energies for TLR5-FAVC-FSE interacting residues following molecular dynamics simulation.

Interacting FAVC residue	Energy (kcal/mol)	Interacting TLR5 residue	Energy (kcal/mol)
PHE 507	–4.146 ± 1.238	TYR1005	–3.622 ± 1.461
TYR 511	–3.698 ± 1.382	TYR 683	–3.022 ± 1.077
VAL 546	–3.587 ± 0.625	PHE 812	–2.753 ± 0.716
PRO 451	–2.194 ± 0.974	PHE 707	–2.723 ± 0.566
ILE 548	–2.051 ± 0.816	HIS 907	–2.437 ± 1.390
PHE 500	–1.662 ± 0.858	LEU 688	–2.320 ± 1.433
GLY 544	–1.653 ± 0.806	ASP1028	–2.186 ± 2.642
CYS 443	–1.526 ± 1.255	GLN1075	–2.143 ± 1.422
LEU 503	–1.518 ± 0.565	TYR 662	–1.996 ± 2.666
ASN 549	–1.378 ± 0.987	GLN 706	–1.975 ± 1.305
LEU 469	–1.361 ± 2.210	ASN 663	–1.921 ± 1.423
TYR 499	–1.329 ± 1.117	TYR 899	–1.848 ± 1.121
LYS 486	–1.327 ± 1.004	ILE 665	–1.744 ± 0.450
CYS 487	–1.267 ± 1.134	HIS1052	–1.725 ± 1.311
MET 496	–1.230 ± 0.849	THR1044	–1.722 ± 1.563
SER 561	–1.225 ± 1.393	GLY1079	–1.692 ± 1.013
CYS 467	–1.164 ± 1.158	ILE 667	–1.605 ± 0.573
ILE 447	–1.114 ± 1.240	ASN 668	–1.515 ± 1.303
LYS 458	–1.100 ± 2.441	ILE 731	–1.466 ± 0.364
ASN 446	–1.089 ± 1.631	SER1080	–1.348 ± 1.352
LEU 504	–1.084 ± 0.581	ILE 732	–1.238 ± 0.352
GLN 454	–1.003 ± 1.780	TRP1050	–1.053 ± 1.185
		THR1029	–1.030 ± 0.679

TLR5 = Toll-like receptor 5; FAVC = Fused antigen vaccine candidate.

**Table 9.** Decomposed binding energies for TLR5-FAVC-CTB interacting residues following molecular dynamics simulation.

Interacting FAVC residue	Energy (kcal/mol)	Interacting TLR5 residue	Energy (kcal/mol)
PHE 185	–5.260 ± 1.490	ARG 686	–4.352 ± 2.970
ASN 213	–5.184 ± 1.287	ARG 739	–3.934 ± 2.959
GLU 234	–4.138 ± 3.593	PHE 582	–3.728 ± 1.076
PHE 178	–3.793 ± 1.781	PRO 773	–3.049 ± 2.492
LEU 225	–3.021 ± 1.699	PHE 660	–2.715 ± 0.487
TYR 189	–2.914 ± 1.502	GLU 636	–2.672 ± 1.873
HIS 214	–2.789 ± 0.783	HIS 584	–2.562 ± 1.274
LYS 313	–1.949 ± 1.860	ASN 522	–2.415 ± 1.174
ASN 175	–1.903 ± 1.175	ILE 771	–2.259 ± 1.776
LEU 182	–1.857 ± 0.530	PHE 587	–2.197 ± 0.870
LEU 310	–1.734 ± 1.371	TYR 524	–1.912 ± 0.873
GLU 228	–1.709 ± 1.484	LEU 526	–1.628 ± 1.238
LEU 192	–1.441 ± 0.587	GLY 762	–1.401 ± 0.780
VAL 224	–1.284 ± 0.687	HIS 684	–1.364 ± 0.925
LEU 188	–1.265 ± 0.575	SER 769	–1.045 ± 1.120
MET 174	–1.138 ± 0.790		
ASN 329	–1.075 ± 1.329		

TLR5 = Toll-like receptor 5; FAVC = Fused antigen vaccine candidate.

**Figure 10.**
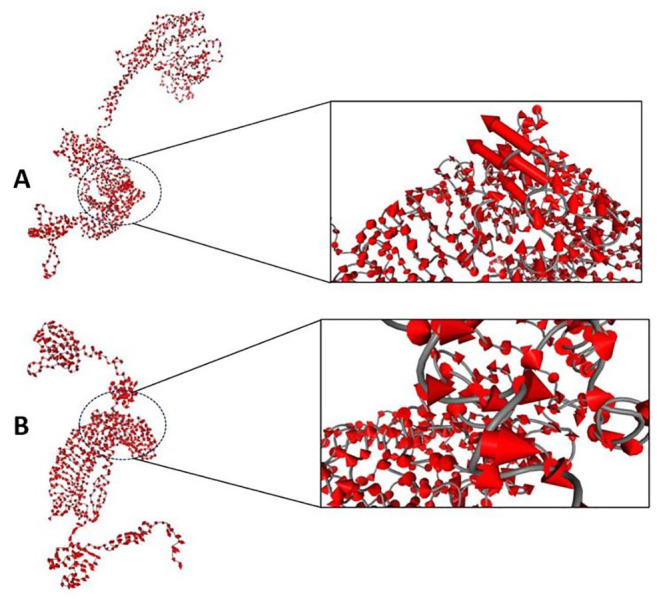
Normal mode analysis mobility of protein domains using MD covariance matrix of (A) TLR5-FAVC-FSE and (B) TLR5-FAVC-CTB. The arrow at each residue represents both the magnitude of motions and the direction of movements of residues with respect to each other.

**Figure 11.**
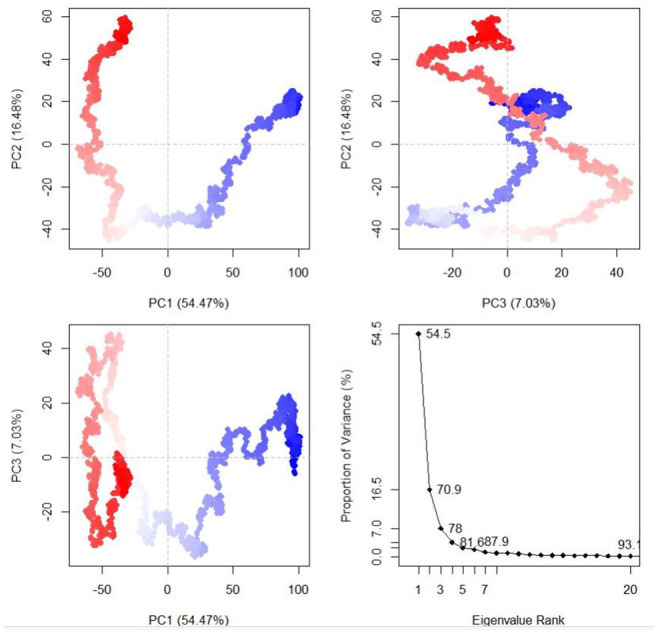
Principal component analysis of TLR5-FAVC-FSE. TLR5 = Toll-like receptor 5; FAVC = Fused antigen vaccine candidate.

**Figure 12.**
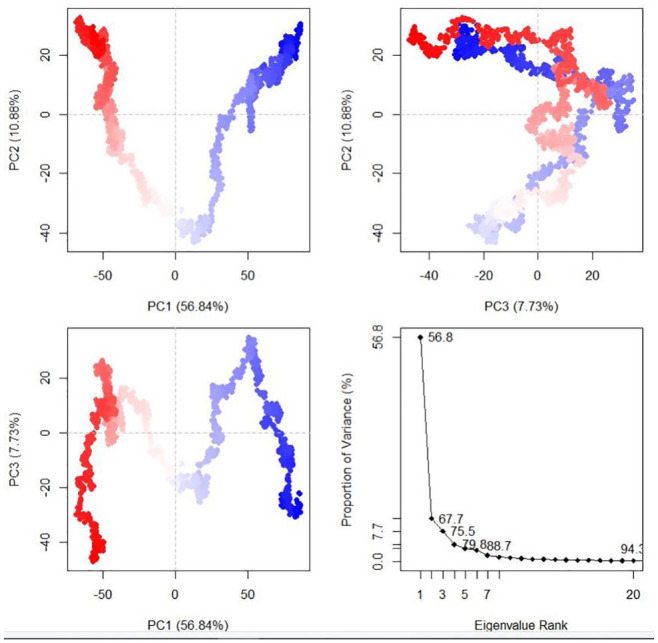
Principal component analysis of TLR5-FAVC-CTB. TLR5 = Toll-like receptor 5; FAVC = Fused antigen vaccine candidate.

**Figure 13.**
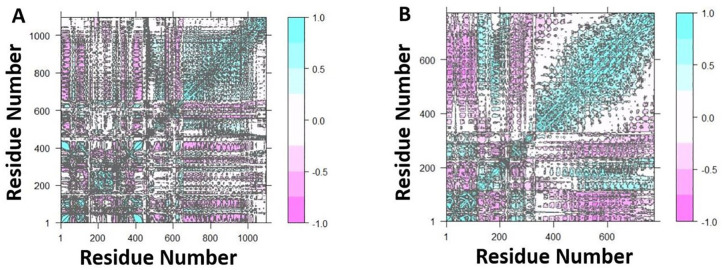
Dynamic cross-correlation plots of (A) TLR5-FAVC-FSE and (B) TLR5-FAVC-CTB. Positive correlation (blue) and negative correlation (pink) based on residue motion. TLR5 = Toll-like receptor 5; FAVC = Fused antigen vaccine candidate.

The dynamics and motions of TLR5-FAVC were further investigated on the ensemble of structures extracted from an equilibrated MD trajectory and these structures represent proteins’ conformational space during the simulation. The collective functional motion and flexibility of TLR5-FAVCs were described through NMA. The arrows in Figure 10 showed that upon binding, the FAVC and the TLR5 were significantly directed to each other^52^ and this mode represents protein-protein interactions. Principal component (PC) analysis plots were used to examine the different conformational changes in the structure of TLR5-FAVC as depicted in Figure 11 for TLR5-FAVC-FSE and Figure 12 for TLR5-FAVC-CTB. A total of 93.1% (TLR5-FAVC-FSE) and 94.3% (TLR5-FAVC-CTB) of the variance of conformational fluctuation was captured for 20 principal components. The contributions of PC1, PC2, and PC3 were 54.47%, 16.48%, and 7.03%, respectively, for TLR5-FAVC-FSE and 56.84%, 10.88%, and 7.73%, respectively, for TLR5-FAVC-CTB. During the simulation, the magnitude of motion to direction was indicated from blue to white and then to red. The dynamic cross-correlation matrix indicates the extent to which the atomic fluctuation of a system is correlated with each other. The blue color represented a positive correlation while the pink represented a negative correlation. The results of cross-correlation analysis as shown in Figure 13 indicated a high positive correlation for TLR5 residues relative to those of FAVC.

## Discussion

The development of the malaria vaccine has taken about 6 decades resulting in the approval of RTS, S/AS01 vaccine in 2021 by the World Health Organization and there is still a need to search for and develop new vaccine candidates against malaria parasites.^60^ Hence, it becomes important to target different developmental stages and possible combinations of antigens of the malaria parasite for vaccine development. Experimental works involving up to 2 fused antigens showed promising immunogenicity for transmission-blocking vaccine development against *Plasmodium falciparum*.^18-21,61^ On fused antigens targeting preerythrocytic malaria, Lu et al^15^ concluded that fusion of circumsporozoite protein and thrombospondin-related adhesion protein increased protein antigen production with the potential to produce effective vaccines. Formulations, opportunities, and challenges in the development of vaccines have been extensively reviewed.^62-64^

In this study, the sexual stage of *P. falciparum* was targeted to improve the transmission-blocking characteristics of the vaccine candidate. The peptides generated from 4 sexual-stage antigens (Pfs25, Pfs28, Pfs48/45, and Pfs230) were fused as it had earlier been reported that fused antigens increased protein antigen production for effective vaccines.^15^ The Pfs48/45 and Pfs230 antigens affect male fertility in *P. falciparum* because the deficiency of these antigens results in the inability of male gametes to recognize and attach to female gametes to form ookinetes.^10,12^ Also, both Pfs25 and Pfs28 play a crucial function in the survival of ookinetes in the mosquito’s gut^11^ and as such elicit antibodies with transmission-reducing activity.

Although the number of sequences retrieved from NCBI varied across the 4 sexual stages of the antigens targeted in this study for vaccine design, the number of unique sequences seemed not to be directly proportionate with the number of sequences retrieved. This indicates from the current work that a high number of sequences in databases does not necessarily translate to a high number of unique sequences as also observed in the study of Ishwarlall et al.^65^ Furthermore, between 66.12% and 100% of the unique sequences passed the first condition of antigenicity test while 65.96% to 100% of these unique antigenic peptides were outside the membrane. First, in general, outside membrane peptides are important for vaccine development because of the exposure of their epitopes outside the cell surface, a trait that enhances their immunogenicity.^66^ Second, transmembrane peptides are highly conserved across different species.^66,67^ Third, Maiti et al^66^ further indicated that outer membrane proteins contain pathogen-associated molecular patterns that are also found on host cells that participate in the uptake, processing, and presentation of antigens to adaptive immune system cells to induce long-term immunity. To narrow down the selection of peptides, only the top best unique antigenic peptides that were nonallergens for each antigen were selected to construct FAVCs. To the end users, the final vaccine consisting of antigen and adjuvant is a very crucial consideration and the most desirable form.^64^ Two vaccine candidates were formulated based on the adjuvants FSE and Cholera toxin B (CTB) to give FAVC-FSE and FAVC-CTB. Many proteins of *Plasmodium* show high levels of polymorphism across different species and strains.^11,68^ This variability complicates vaccine development against malaria parasites. Therefore, one of the key considerations in malaria vaccine development is the broad protection between species and strains of *Plasmodium*.^69^ In terms of similarity search of the predicted peptides and the constructed fused antigens against other species of *Plasmodium*, the low E-values reported in this study signify the high conservancy of these peptides with other *Plasmodium* species. This suggested the possibility of the vaccines constructed from the predicted peptides to provide protection across multiple species of *Plasmodium*, thus overcoming antigenic diversity which is one of the major hurdles in malaria vaccine development.^70^

The molecular weight of 70.06 and 37.12 kDa obtained for FAVCs were within the range for an efficient lymphatic system uptake for a vaccine formulation.^71^ The theoretical isoelectric point (*pI*) between 5.00 and 6.37 for FAVCs in this study indicates that they are acidic, and these are the pH at which these constructs have a net charge of zero because negative and positive charges are at equilibrium.^72^ The molecular weight of FAVC-FSE almost doubled that of FAVC-CTB. From the work of Atapour et al^73^ on the computational design of a multiepitope vaccine against blood-stage malaria infection, the molecular weight of FAVC-FSE (70.06 kDa) in the present study was higher than 46 kDa reported. The theoretical isoelectric points were within the range of a native protein. The *pI* will play an important role in determining the biochemical function of FAVC because solubility and electrical repulsion are lowest at *pI*.^74^ The time it takes for half of a protein to decay after production in a cell is known as its half-life.^73^ The amino acid of the N-terminal of a protein determines its half-life and the N-terminal of FAVCs sequences is glycine.^75^ The half-life of 30 and 7.2 hours in mammalian reticulocytes reported for FAVCs in this investigation was 85.33% (FAVC-FSE) and 38.89% (FAVC-CTB) higher than the 4.4 hours obtained by Maharaj et al,^76^ for a multiepitope vaccine against malaria *P. falciparum* although different antigens were used. Since the instability indexes of 17.42 and 33.44 are less than 40, it indicates that it is low and therefore shows that FAVCs are stable. FAVC-FSE had higher stability compared with the result of Maharaj et al,^76^ which reported an instability index of 30.28 in a multiepitope vaccine design against *P. falciparum*. The solubility of FAVC is predicted by the direction of the GRAVY value. In this study, the GRAVY values of FAVCs were negative indicating their solubility in water is hydrophilic. It is also an indication that the hydrophilic area of the FAVCs is surface exposed, making them suitable for immune response recognition. The positive and high values of the aliphatic index for FAVCs show that they are thermostable. The interaction of the vaccine with the immune cells can be significantly influenced by the net charge.^77^ The net charge of the predicted FAVCs is negative indicating more attraction for a positively charged immune host. The negative net charge is confirmed by the values of the *pI* and higher negatively charged residues present in the vaccine constructs.^78^ The value of the extended strand obtained for FAVCs is about the average expected to contribute to the stability of the secondary and resulting tertiary structure of the construct.^79^ Although the percentages of beta turns obtained in this study are low, it shows the presence of beta turns in the vaccine constructs (FAVCs). Working with pathogenic *P. falciparum*, it was concluded that long-lasting protective immunity-like structures must precede beta turns to induce long-lasting protective immunity with immune protection-inducing protein structures.^80^ Among the nonregular structure parameters, the random coil has the highest percentage representation ranging between 29.92% and 51.05%. It can be deduced from the beta turns and random coils the ease of forming the epitopes, particularly any construct with a high proportion of beta turns and random coils to have a good structural basis for vaccine design.^81^ The contents of the secondary structures obtained in the present study are not too far from the ones obtained for *P. falciparum* in another study.^82^ The negative values obtained for the *z*-score are an indication that the tertiary structures of FAVCs are good models.^83^ From the Ramachandra plot, most residues were found in the favored regions and this, therefore, indicates the good quality of FAVCs. This is also further supported by the values for the overall quality of FAVCs. The results of 94.20% and 89.85% from Errat analysis show a high resolution of the correct distribution of atoms in FAVC.^33^ The overall quality of the FAVC-FSE shows better results relative to the Ramachandran plot of 90.90% obtained by Pandey et al,^84^ while that of FAVC-CTB was lower.

The antigenicity score of FAVCs is an indication of their ability to bind to B-cell receptors so that the host can elicit antibody responses. The antigenicity scores in the current study are lower than that of Maharaj et al,^76^ with a score of 1.1810 but relatively higher than that of Pandey et al,^84^ that obtained 0.5247. In malaria vaccines, the identification of T-cell epitopes in the antigenic proteins that can effectively bind to HLA is important.^85^ The presence of CD8^+^ and CD4^+^ in the vaccine candidates ensures a stronger immune response in the body. Memory B-cells help in eliciting immune response against *P. falciparum* infection. According to Ayieko et al,^86^ there was an association between a 12-month nondetectable *P falciparum* infection with large changes in overall B-cell memory subsets in the Kipsamoite and Kapsisiywa areas of Kenya. In another study in Kenya, stable memory B-cells were documented in children with previous *P. falciparum*-specific infection without subsequent exposure.^87^ Following up a cohort of people with malaria infection in the last 6 years for 12 months, antibodies were detected and confirmed the establishment of memory B-cells that were stable for many years against the malaria parasite.^88^ The conformational B-cells of FAVCs scores show that they have antigenic determinants with residues from different sections of sequences to fold to their native structure.^89^ The ability of the vaccine candidates to clear the antigen on secondary exposure by eliciting a specific immune response was investigated by the in silico immune simulation. The steady rise of the antibodies as well as the increase in the T-cell populations with the repeated exposure to the vaccine construct confirmed the ability of the FAVCs to trigger an immune response when delivered into the living systems.

Toll-like receptors play crucial roles in pathogen detection and inflammatory response initiation.^90^ In a study conducted by Bargieri et al,^91^ it was concluded that the fusion of the innate immunity agonist *Salmonella enterica* serovar Typhimurium flagellin would improve their immunogenic responses and that the binding of bacterial flagellins to extracellular TLR5 and intracellular receptors lead to strong inflammatory response as summarized by Bargieri et al.^91^ In the construction of FAVCs, the adjuvants used were FSE and Cholera Toxi B. Molecular docking of TLR5 with FAVCs was carried out to determine their binding interactions. Signals through TLR5 result in the upregulation of costimulatory molecules that are needed for the activation and interaction of dendritic and T-cells to begin the process of adaptive immune responses.^92^ A strong inflammatory response resulted from the binding of bacterial flagellins, especially the ones expressed by *Salmonella* species as documented in a study.^91^ Cholera Toxi B enhances the immune response to antigen.^93^ The 18 and 16 (TLR5-FAVC-FSE and TLR5-FAVC-CTB) hydrogen bonds present in the analysis of TLR5-FAVCs help to stabilize each loop in the complex. As indicated by Collins et al,^94^ hydrophobic interactions play critical roles in the formation of protein-protein complex formation. The TLR5-FAVCs contained 89 and 73 (TLR5-FAVC-FSE and TLR5-FAVC-CTB) hydrophobic interactions. The negative and high binding energies of -67.47 and -69.14 kcal/mol (TLR5-FAVC-FSE and TLR5-FAVC-CTB) from MD simulation is an indication of a good binding affinity in the TLR5-FAVC complexes. From the result of MD simulations, peptides P1 and P3 (Pfs25 and Pfs28, respectively) were mostly involved in the interactions for FAVC-FSE while P3 and P10 (Pfs28 and Pfs230, respectively) for FAVC-CTB. Pfs48/45 is hardly involved in the interaction. The change in color in the principal component analysis from blue to white and from white to red does indicate periodic jumps between different conformations throughout the trajectory during MD simulation. The residue motions of the vaccine construct had both negative and positive correlations. Cross-correlation estimates the correlation coefficient of the motions between the atoms of TLR5 and FAVCs.

## Conclusion

To the best of our knowledge, this study is the first immunoinformatics investigation that fused 4 sexual-stage antigens (Pfs25, Pfs28, Pfs48/45, and Pfs230) for a transmission-blocking malaria vaccine. The generated FAVCs, FAVC-FSE and FAVC-CTB, demonstrated promising antigenic, immunogenic, nonallergenic, and stable properties. The study highlights the potential of these FAVCs to elicit strong immune responses, which are crucial for breaking the transmission cycle of malaria. The molecular docking and dynamics simulations further confirmed the strong binding interactions between the FAVCs and Toll-like receptor 5 (TLR5), indicating their potential effectiveness in triggering immune responses. The overall quality of FAVC-FSE is better than that of FAVC-CTB except in RMSD analysis in which the latter is better. Despite promising computational results, laboratory validation is still needed to confirm the predicted characteristics and ensure the efficacy of these vaccine candidates. Overall, this research provides a foundation for the development of a novel transmission-blocking malaria vaccine, with implications for malaria control, particularly in endemic regions.

## Supplemental Material

sj-docx-1-bbi-10.1177_11779322241306215 – Supplemental material for Computational Development of Transmission-Blocking Vaccine Candidates Based on Fused Antigens of Pre- and Post-fertilization Gametocytes Against Plasmodium falciparum Supplemental material, sj-docx-1-bbi-10.1177_11779322241306215 for Computational Development of Transmission-Blocking Vaccine Candidates Based on Fused Antigens of Pre- and Post-fertilization Gametocytes Against Plasmodium falciparum by Matthew A. Adeleke in Bioinformatics and Biology Insights
