# Supplementary material for: Computational Development of Transmission-Blocking Vaccine Candidates Based on Fused Antigens of Pre- and Post-fertilization Gametocytes Against Plasmodium falciparum
Source: Bioinform Biol Insights. 2025 Mar 3;19:11779322241306215. doi: 10.1177/11779322241306215 (PMC11873872; doi:10.1177/11779322241306215)
Supplement: sj-docx-1-bbi-10.1177_11779322241306215 – Supplemental material for Computational Development of Transmission-Blocking Vaccine Candidates Based on Fused Antigens of Pre- and Post-fertilization Gametocytes Against Plasmodium falciparum [file sj-docx-1-bbi-10.1177_11779322241306215.docx]

**Table S1**: Accession number of sequences downloaded from blast search

| Pfs25 | Pfs28 | Pfs48/45 | Pfs230 |
| --- | --- | --- | --- |
| KNG76875.1 | AAT00624.1 | AAL74353.1 | AAA29734.1 |
| UFQ05501.1 | XP_001347586.1 | AAL74354.1 | AAG12332.1 |
| UFQ05505.1 | | AAL74355.1 | UIT08942.1 |
| UFQ05506.1 | | AAL74356.1 | UIT08905.1 |
| UFQ05509.1 | | AAL74352.1 | UIT08907.1 |
| UFQ05511.1 | | AAL74337.1 | UIT08908.1 |
| UFQ05523.1 | | AAL74338.1 | UIT08909.1 |
| UFQ05524.1 | | AAL74345.1 | UIT08912.1 |
| UFQ05532.1 | | AAL74346.1 | UIT08913.1 |
| UFQ05539.1 | | AAL74351.1 | UIT08915.1 |
| UFQ05541.1 | | AAL74357.1 | UIT08918.1 |
| UFQ05542.1 | | AAL74347.1 | UIT08921.1 |
| UFQ05564.1 | | AAL74350.1 | UIT08925.1 |
| UFQ05490.1 | | AAL74360.1 | UIT08927.1 |
| UFQ05491.1 | | AAL74340.1 | UIT08928.1 |
| UFQ05492.1 | | AAL74341.1 | UIT08933.1 |
| UFQ05493.1 | | AAL74342.1 | UIT08934.1 |
| UFQ05496.1 | | AAL74343.1 | UIT08935.1 |
| UFQ05497.1 | | AAL74348.1 | UIT08939.1 |
| UFQ05498.1 | | AAL74359.1 | UIT08943.1 |
| UFQ05500.1 | | AAL74361.1 | UIT08944.1 |
| UFQ05502.1 | | AAL74344.1 | UIT08945.1 |
| UFQ05503.1 | | AAL74362.1 | UIT08946.1 |
| UFQ05504.1 | | AAL74363.1 | UIT08947.1 |
| UFQ05507.1 | | AAL74364.1 | UIT08948.1 |
| UFQ05508.1 | | AAL74365.1 | UIT08949.1 |
| UFQ05510.1 | | AAL74366.1 | UIT08950.1 |
| UFQ05512.1 | | AAL74367.1 | UIT08951.1 |
| UFQ05513.1 | | AAL74368.1 | UIT08952.1 |
| UFQ05514.1 | | AAL74369.1 | UIT08953.1 |
| UFQ05515.1 | | AAL74370.1 | UIT08954.1 |
| UFQ05516.1 | | AAL74371.1 | UIT08955.1 |
| UFQ05517.1 | | AAL74372.1 | UIT08956.1 |
| UFQ05518.1 | | AAL74373.1 | UIT08957.1 |
| UFQ05519.1 | | AAL74374.1 | UIT08958.1 |
| UFQ05520.1 | | AAL74375.1 | UIT08962.1 |
| UFQ05521.1 | | AAL74376.1 | UIT08979.1 |
| UFQ05526.1 | | AAL74378.1 | UIT08980.1 |
| UFQ05527.1 | | AAL74339.1 | UIT08981.1 |
| UFQ05528.1 | | AAL74349.1 | UIT08983.1 |
| UFQ05529.1 | | AAL74358.1 | UIT08984.1 |
| UFQ05530.1 | | AAL74379.1 | UIT08985.1 |
| UFQ05531.1 | | AAL74377.1 | UIT08986.1 |
| UFQ05533.1 | | AAL74380.1 | UIT08988.1 |
| UFQ05534.1 | | ABO41492.1 | UIT08992.1 |
| UFQ05535.1 | | ABO41489.1 | UIT08994.1 |
| UFQ05536.1 | | ABO41497.1 | UIT08995.1 |
| UFQ05537.1 | | ABO41490.1 | UIT08998.1 |
| UFQ05540.1 | | ABO41491.1 | UIT09000.1 |
| UFQ05543.1 | | ABO41493.1 | UIT09001.1 |
| UFQ05544.1 | | ABO41494.1 | UIT09002.1 |
| UFQ05545.1 | | ABO41495.1 | UIT09003.1 |
| UFQ05546.1 | | ABO41496.1 | UIT09009.1 |
| UFQ05547.1 | | ASU08935.1 | UIT09011.1 |
| UFQ05548.1 | | ASU08937.1 | UIT09012.1 |
| UFQ05550.1 | | ASU08936.1 | UIT09013.1 |
| UFQ05551.1 | | AHA91190.1 | UIT09018.1 |
| UFQ05552.1 | | AHA91189.1 | UIT09019.1 |
| UFQ05553.1 | | AHB33180.1 | UIT09021.1 |
| UFQ05554.1 | | AHB33182.1 | UIT09022.1 |
| UFQ05555.1 | | AHB33181.1 | UIT09025.1 |
| UFQ05556.1 | | UIT09071.1 | UIT09026.1 |
| UFQ05558.1 | | UIT09072.1 | UIT09028.1 |
| UFQ05559.1 | | UIT09073.1 | UIT09030.1 |
| UFQ05561.1 | | UIT09074.1 | UIT09031.1 |
| UFQ05562.1 | | UIT09075.1 | UIT09032.1 |
| UFQ05563.1 | | UIT09076.1 | UIT09033.1 |
| UFQ05565.1 | | UIT09077.1 | UIT09034.1 |
| UFQ05566.1 | | UIT09078.1 | UIT09035.1 |
| UFQ05567.1 | | UIT09079.1 | UIT09037.1 |
| UFQ05568.1 | | UIT09080.1 | UIT09038.1 |
| UFQ05569.1 | | UIT09081.1 | UIT09039.1 |
| UFQ05570.1 | | UIT09082.1 | UIT09042.1 |
| AAF63684.1 | | UIT09083.1 | UIT09043.1 |
| AAD39544.1 | | UIT09084.1 | UIT09044.1 |
| AAD55785.1 | | UIT09085.1 | UIT09045.1 |
| KNC37697.1 | | UIT09086.1 | UIT09049.1 |
| UFQ05522.1 | | UIT09087.1 | UIT09051.1 |
| UFQ05525.1 | | UIT09088.1 | UIT09052.1 |
| UFQ05538.1 | | UIT09089.1 | UIT09054.1 |
| UFQ05549.1 | | UIT09090.1 | UIT09055.1 |
| UFQ05557.1 | | UIT09091.1 | UIT09056.1 |
| UFQ05560.1 | | UIT09092.1 | UIT09057.1 |
| UFQ05488.1 | | UIT09093.1 | UIT08940.1 |
| UFQ05489.1 | | UIT09094.1 | UIT08941.1 |
| UFQ05494.1 | |  | UIT08960.1 |
| UFQ05495.1 | |  | UIT09029.1 |
| UFQ05499.1 | |  | UIT08906.1 |
|  |  |  | UIT08910.1 |
|  |  |  | UIT08914.1 |
|  |  |  | UIT08916.1 |
|  |  |  | UIT08919.1 |
|  |  |  | UIT08920.1 |
|  |  |  | UIT08922.1 |

**Table S2**: Pfs25 peptides along with its antigenicity score

| Peptide | Antigenic score |
| --- | --- |
| ILDTSNPVKTGVCSCNIGKVPNVQDQNKCSKDGETKCSLKCLKENETCKAVDGIYKCDCK | 0.8409 |
| ILDTSNPVKTAVCSCNIGKVPNAQDQNKCSKDGETKCSLKCLKENETCKAVDGIYKCDCK | 0.8245 |
| MNKLYSLFLFLFIQLSIKYNNAKVTVDTVCKRGFLIQMSGHLECKCENDLVLVNEETCEE | 0.8223 |
| ILDTSNPVKTAVCSCNIGKVPNVQDQNKCSKDGETKCSLKCLKENETCKAVDGIYKCDCK | 0.7776 |
| ILDTSNPVKTAVCSCNIGKVPNVQNQNKCSKDGETKCSLKCLKENETCKAVDGIYKCDCK | 0.7589 |
| KVLKCDEKTVNKPCGNFSKCIKIDGNPVSYACKCNLGYDMVNNVCIPNECKNVTCGNGKC | 0.7213 |
| KVLKCDEKTVNKPCGDFSKCIKIDGNPVSYACKCNLGYDMVNNVCIPNECKNVTCGNGKC | 0.7189 |
| DGFIIDNESSICTAFSAYNILNLSIMFILFSVCFFIM | 0.5775 |

**Table S3**: Pfs28 peptides along with its antigenicity score

| Peptide | Antigenic score |
| --- | --- |
| MNTYFKVLLFLFIQLYITLNKARVTENTICKYGYLIQMSNHYECKCIEGYVLINEDTCGK | 0.9098 |
| KCIVDPANSLTHTCSCNIGTILNQNKLCDIQGDTPCSLKCAENEVCTLEGNYYTCKEDPS | 0.5983 |
| SNGGGNTVDQADTSYSVINGVTLTHVLIVCSIFIKLLI | 0.5793 |

**Table S4**: Pfs48/45 peptides along with its antigenicity score

| Peptide | Antigenic score |
| --- | --- |
| MTVTIDSAYYGFLAKTFILLIVAILLYI | 1.4000 |
| MTVTIDSAYYGFLAKTFIFLIVAILLYI | 1.3421 |
| MIVTIDSAYYGFLAKTFIFLIVAILLYI | 1.2250 |
| MTVTIDSAYYGFLAKTFIFLIV | 1.2131 |
| MTVTIDSAYYGFLAKTFIFLIVAI | 1.2023 |
| SSNDSSKHTFTDSLDISLVDDNAHISCNVHLSEPKYNHLVGLNCPGDIVPDCFFLVYQPE | 1.0393 |
| SSNDSSKHTFTDSLDISLVDDNAHISCNVHLSEPKYNHLVGLNCPGDIIPDCFFQVYQPE | 1.0172 |
| SSNDSSKHTFTDSLDISLVDDSAHISCNVHLSEPKYNHLVGLNCPGDIIPDCFFQVYQPE | 1.0017 |
| SSNVSSKHTFTDSLDISLVDDSAHISCNVHLSEPKYNHLVGLNCPGDIIPDCFFQAYQPE | 0.9944 |
| SSNVSSKHTFTDSLDISLVDDSAHISCNVHLSGPKYNHLVGLNCPGDIIPDCFFQVYQPE | 0.9923 |
| SSNVSSKHTFTDSLDISLVDDCAHISCNVHLSEPKYNHLVGLNCPGDIIPDCFFQVYQPE | 0.9627 |
| SSNVSSKHTFTDSLDISLVDDNAHISCNVHLSEPKYNHLVGLNCPGDIIPDCFFQVYQPE | 0.9557 |
| SSNVSSKHTFTDSIDISLVDDSAHISCNVHLSEPKYNHLVGLNCPGDIIPDCFFQVYQPE | 0.9438 |
| SSNVSSKHTFTDSLDISLVDDSAHISCNVHLSEPKYNHLVGLNCPGDIIPDCFFQVYQPE | 0.9402 |
| SSNVSSKHTFTDSLDISLVDDNAHISCNVHLSEPKYNHLVGLNCPGDIVPDCFFQVYQPE | 0.9288 |
| SSNVSSKHTFTDGLGISLVDDSAHISCNVHLSEPKYNHLVGLNCPGDIIPDCFFQVYQPE | 0.8202 |
| KALYKSNKIIYHKKLTIFKAPFYVTSKDVNTECTCKFKNNNYKIVLKPKYEKKVIHGCNF | 0.8001 |
| KALYKSNKIIYHEKLTIFKAPFYVTSKDVNTECTCKFKNNNYKIVLKPKYEKKVIHGCNF | 0.7967 |
| KALYKSNKIIYHNKLTIFKAPFYVTSKDVNTECTCKFKNNNYKIVLKPKYEKKVIHGCNF | 0.7830 |
| KALYKSNKIIYHKNLTIFKAPFYVTSKDVNTECTCRFKNNNYKIVLKPKYEKKVIHGCNF | 0.7800 |
| KALYKSNKIIYHKNLTIFKAPFYVTSKDVNTECTCKFKNNNYKIVLKPKYEKKVIHGCNF | 0.7773 |
| KYPHNILFTNLTNDLFTYLPKTYNESNFVSNVLEVELNDGELFVLACELINKKCFQEGKE | 0.6640 |
| MMLYISAKKAQVAFILYIVLVLRIISGNNDFYKPSSLNSEISGFIGYKCNFSNEGVHNLK | 0.6462 |
| MMLYISAKKAQVAFILYIVLVLRIISGNNDFYNPSALNSEISGFIGYKCNFSNEGVHNLK | 0.6224 |
| MMLYISAKKAQVAFILYIVLVLRIISGNNDFCKPSSLNSEISGFIGYKCNFSNEGVHDLK | 0.6155 |
| KKAQVAFILYIVLVLRIISGNNDFCKPSSLNSEISGFIGYKCNFSNEGVHNLK | 0.5934 |
| MMLYISAKKAQVAFILYIVLVLRIISGNNDFCKPSSLNSEISGFIGYKCNFSNEGVHNLK | 0.5877 |
| DNTEKVISSIEGRSAMVHVRVL | 0.5718 |
| SEELEPSNIVYLDSQINIGDIEYYEDAEGDDKIKLFGIVGSIPKTTSFTCICKEDKKSAY | 0.5415 |
| LGLIEYEIEENDTNPNYNERTITISPFSPKDLEFFCFCDNTEKVISSIEGRSAMVHVRVL | 0.5335 |
| LGLIEYEIEENDTNPNYNERTITISPFSPKDIEFFCFCDNTEKVISSIEGRSAMVHVRVL | 0.5288 |
| LGLIEYEIEENDTNPNYNERTLTISPFSPKDIEFFCFCDNTEKVISSIEGRSAMVHVRVL | 0.5253 |
| SEELEPSNIVYLDSQINIGDIEYYEDAEGDDKIKLFGIVGSIPKTTSFTCICKKDKKS | 0.5143 |
| LGLIEYEIEENDTNPNYNERTITISPFSPEDIEFFCFCDNTEKVISSIEGRSAMVHVRVL | 0.5133 |

**Table S5**: Pfs230 peptides along with its antigenicity score

| Peptide | Antigenic score |
| --- | --- |
| TKLKEKLLSKLIYGLLISPTVNEKENNFKE | 1.2430 |
| TKLKENLLSKLIYGLLISPTVNEKENNFKE | 1.1821 |
| KYALLLDDEERQKKKNQQEEQQEQQEQQEQILKDQDDRLSRHDDYNKNHTYILYDSNEHI | 1.0031 |
| KYALLLDDEERQKKIKQQQEEEQQEQILKDQDDRLSRHDDYNKNHTYILYDSNEHI | 0.9772 |
| MKKIITLKNLFLIILVYIFSEKKDLRCNVIKGNNIKDDEDKRFHLFYYSHNLFKTPETKE | 0.9348 |
| EEEEYDDYVYEESGDETEEQLQEEHQEEVGAESSEESFNDEDEDSVEARDGDMIR | 0.9218 |
| VDTGPVLDNSTFEKYFKNIKIKPDKFFEKVINENDDTEEEKDLESILPGAIVSPMKVLKK | 0.9154 |
| VEEGVQNEEYKKFSLKPSLVFDDNNNDIKVIGKEKNEVSISLALKGVYGNRIFTFDKNGK | 0.9056 |
| EEEEEYDDYVYEESGDETEEQLQEEHQEEVGAESSEESFNDEDEDSVEARDGDMIRVDEY | 0.8761 |
| EEGEEVGEGVGEEVGEEEGEEVGEEEGEYVDEKERQGEIYPFGDEEEKDEGGESFTYEKS | 0.8437 |
| VDTGPVLDNSTFEKYFKNIKIKPDKFFEKVINEYDDTEEEKDLESILPGAIVSPMKVLKK | 0.7809 |
| LYKKLNIKFDTYVTGTDQNKYLMTYMDLHLSHKRNYLKELFHDLGKKKPADTDANPESII | 0.7350 |
| KIKGCNFHESKLDYFNENISSDTHECTLHAYENDIIGFNCLETTHPNEVEVEVEDAEIYL | 0.6840 |
| LKCNVNKTQYPNIEIFPKTLKDKKEVLKLDLDIQYQMFSKFFKFNTQNAKYLNLYPYYLI | 0.6814 |
| SLKYIYMYLTPSDSFNLVRRNRNLDEEDMSPRDNFVMDDEEEEEEEEEEEEEEEEEEEEE | 0.6713 |
| NNIYPCYMKLYSGDIGGILFPKNIKSTTCFEEMIPYNKEIKWNKENKSLGNLVNNSVVYN | 0.6697 |
| SLKYIYMYLTPSDSFNLVRRNRNLDEEDMSPRDNFVIDDEEEEEEEEEEEEEEEEEEEEE | 0.6664 |
| VVKKIKVIITKNDTVLLKREVQSESTLDDKIYKCEHENFINPRVNKTFDENVEYTCNIKI | 0.6466 |
| EVDKTDLFKFIEGGEGDDVYKVDGSKVLLDDDTISRVSKKHTARDGEYGEYGEAVEDGEN | 0.6389 |
| LYKKLNIKFDNYVTGTDQNKYLMTYMDLHLSHKRNYLKELFHDLGKKKPADTDANPESII | 0.6389 |
| TKLKEKLLSKLIYGLLISPTVNEKENNFKEGVIEFTLPPVVHKATVFYFICDNSKTEDDN | 0.6346 |
| HNVLHNVVLNNHIVNLSSALEGVLFMKSKVTGDETATKKNTTLPTDGVSSILIPPYVKED | 0.6283 |
| YEDQDGDTYDSTIKNEDVDEDADEEVGEEVGEEVGEEVGEEVGEEVGEEVGEEVGEEVGE | 0.6238 |
| FSTNNSSILTSSVKLVNGETKNCEININNNEVFGIICDNETNLDPEKCFHEIYSKDNKTV | 0.6223 |
| YSTNNSSILTSSVELVNGETKNCEININNNEVFGIICDNETNLDPEKCFHEIYSKDNKTV | 0.6130 |
| YENKDYESDDKLIEWFDDNTNEENFLLTFLKRCLMKIFSSPKRKKTVVQKKHKSNFFINS | 0.6047 |
| LQVNLKAPKLMMSAQIHNNRHVCDFSKNNLIVPESLKKKEELGGNPVNIHCYALLKPLDT | 0.5992 |
| YENKDYESDDTLIEWFDDNTNEENFLLTFLKRCLMKIFSSPKRKKTVVQKKHKSNFFINS | 0.5963 |
| PQSRAIIKYVDLQDKNFAKYLRKL | 0.5911 |
| CDYEKNEPLISTLPNDTKKIQKSICKINAKALDVVTIKCPHTKNFTPKDYFPNSSLITND | 0.5700 |
| KDPFTSYAAFVVPPIVPKDLHFKVECNNTEYKDENQYISGYNGIIHIDISNSNRKINGCD | 0.5602 |
| EKEEEDFKTAQGIKHNNVHLCNFFDNPELTFDNNKIVLCKIDAELFSEVIIQLPIFGTKN | 0.5492 |
| CDYEKNESLISTLPNDTKKIQKSICKINAKALDVVTIKCPHTKNFTPKDYFPNSSLITND | 0.5489 |
| VDEYYEDQDGDTYDSTIKNEDVDEEVGEEVGEEVGEEVGEEVGEEVGEEVGEEVGEEVGE | 0.5194 |
| LYVKCPTSKDNYEAAKVNISENDNEYELQVISLIEKRFHNFETLESKKPGNGDVVVHNGV | 0.5097 |
| YNTFSKDKIGNILKNAISINNPDEKDNTYTYLILPEKFEEELIDTKKVLACTCDNKYIIH | 0.5016 |

**Table S6**: Topology results of the peptide that were outside membrane for Pfs25

| Peptide |
| --- |
| MNKLYSLFLFLFIQLSIKYNNAKVTVDTVCKRGFLIQMSGHLECKCENDLVLVNEETCEE |
| ILDTSNPVKTGVCSCNIGKVPNVQDQNKCSKDGETKCSLKCLKENETCKAVDGIYKCDCK |
| DGFIIDNESSICTAFSAYNILNLSIMFILFSVCFFIM |

**Table S7**: Topology results of the peptide that were outside membrane for Pfs28

| Peptide |
| --- |
| MNTYFKVLLFLFIQLYITLNKARVTENTICKYGYLIQMSNHYECKCIEGYVLINEDTCGK |
| KCIVDPANSLTHTCSCNIGTILNQNKLCDIQGDTPCSLKCAENEVCTLEGNYYTCKEDPS |
| SNGGGNTVDQADTSYSVINGVTLTHVLIVCSIFIKLLI |

**Table S8**: Topology results of the peptide that were outside membrane for Pfs48/45

| Peptide |
| --- |
| KKAQVAFILYIVLVLRIISGNNDFCKPSSLNSEISGFIGYKCNFSNEGVHNLK |
| KYPHNILFTNLTNDLFTYLPKTYNESNFVSNVLEVELNDGELFVLACELINKKCFQEGKE |
| LGLIEYEIEENDTNPNYNERTLTISPFSPKDIEFFCFCDNTEKVISSIEGRSAMVHVRVL |
| MIVTIDSAYYGFLAKTFIFLIVAILLYI |
| MMLYISAKKAQVAFILYIVLVLRIISGNNDFYNPSALNSEISGFIGYKCNFSNEGVHNLK |
| MTVTIDSAYYGFLAKTFIFLIVAILLYI |
| MTVTIDSAYYGFLAKTFILLIVAILLYI |
| SEELEPSNIVYLDSQINIGDIEYYEDAEGDDKIKLFGIVGSIPKTTSFTCICKEDKKSAY |
| SEELEPSNIVYLDSQINIGDIEYYEDAEGDDKIKLFGIVGSIPKTTSFTCICKKDKKS |
| SSNDSSKHTFTDSLDISLVDDNAHISCNVHLSEPKYNHLVGLNCPGDIIPDCFFQVYQPE |
| SSNDSSKHTFTDSLDISLVDDNAHISCNVHLSEPKYNHLVGLNCPGDIVPDCFFLVYQPE |
| SSNVSSKHTFTDGLGISLVDDSAHISCNVHLSEPKYNHLVGLNCPGDIIPDCFFQVYQPE |
| SSNVSSKHTFTDSIDISLVDDSAHISCNVHLSEPKYNHLVGLNCPGDIIPDCFFQVYQPE |
| SSNVSSKHTFTDSLDISLVDDCAHISCNVHLSEPKYNHLVGLNCPGDIIPDCFFQVYQPE |
| SSNVSSKHTFTDSLDISLVDDNAHISCNVHLSEPKYNHLVGLNCPGDIIPDCFFQVYQPE |
| SSNVSSKHTFTDSLDISLVDDNAHISCNVHLSEPKYNHLVGLNCPGDIVPDCFFQVYQPE |
| SSNVSSKHTFTDSLDISLVDDSAHISCNVHLSEPKYNHLVGLNCPGDIIPDCFFQAYQPE |
| SSNVSSKHTFTDSLDISLVDDSAHISCNVHLSEPKYNHLVGLNCPGDIIPDCFFQVYQPE |
| SSNVSSKHTFTDSLDISLVDDSAHISCNVHLSGPKYNHLVGLNCPGDIIPDCFFQVYQPE |

**Table S9**: Topology results of the peptide that were outside membrane for Pfs230

| Peptide |
| --- |
| CDYEKNEPLISTLPNDTKKIQKSICKINAKALDVVTIKCPHTKNFTPKDYFPNSSLITND |
| CDYEKNESLISTLPNDTKKIQKSICKINAKALDVVTIKCPHTKNFTPKDYFPNSSLITND |
| EEEEEYDDYVYEESGDETEEQLQEEHQEEVGAESSEESFNDEDEDSVEARDGDMIRVDEY |
| EEGEEVGEGVGEEVGEEEGEEVGEEEGEYVDEKERQGEIYPFGDEEEKDEGGESFTYEKS |
| EKEEEDFKTAQGIKHNNVHLCNFFDNPELTFDNNKIVLCKIDAELFSEVIIQLPIFGTKN |
| EVDKTDLFKFIEGGEGDDVYKVDGSKVLLDDDTISRVSKKHTARDGEYGEYGEAVEDGEN |
| FSTNNSSILTSSVKLVNGETKNCEININNNEVFGIICDNETNLDPEKCFHEIYSKDNKTV |
| HNVLHNVVLNNHIVNLSSALEGVLFMKSKVTGDETATKKNTTLPTDGVSSILIPPYVKED |
| ITFHLFCGKSTTKKPNKKNTSLALIHIHISSNRNIIHGCDFLYLENQTNDAISNNNNNSY |
| KDPFTSYAAFVVPPIVPKDLHFKVECNNTEYKDENQYISGYNGIIHIDISNSNRKINGCD |
| KGEGISFFIPPIKQDTDLKFIINETIDNSNIKQRGLIYIFVRKNVSENSFKLCDFTTGST |
| KIKGCNFHESKLDYFNENISSDTHECTLHAYENDIIGFNCLETTHPNEVEVEVEDAEIYL |
| KYALLLDDEERQKKIKQQQEEEQQEQILKDQDDRLSRHDDYNKNHTYILYDSNEHI |
| KYALLLDDEERQKKKNQQEEQQEQQEQQEQILKDQDDRLSRHDDYNKNHTYILYDSNEHI |
| LQVNLKAPKLMMSAQIHNNRHVCDFSKNNLIVPESLKKKEELGGNPVNIHCYALLKPLDT |
| LYKKLNIKFDNYVTGTDQNKYLMTYMDLHLSHKRNYLKELFHDLGKKKPADTDANPESII |
| LYKKLNIKFDTYVTGTDQNKYLMTYMDLHLSHKRNYLKELFHDLGKKKPADTDANPESII |
| MLILYNEEKVDLLHFYVFLPIYIKDIYEFNIVCDNSKTMWKNQLGGKVIYHITVSKREQK |
| RYLNLSINELGSDNNTFSVTFQVPPYIDIKEPFYFMFGCNNNKGEGNIGIVELLISKQEE |
| RYLNLSMNELGSDNNTFSVTFQVPPYIDIKEPFYFMFGCNNNKGEGNIGIVELLISKQEE |
| SLKYIYMYLTPSDSFNLVRRNRNLDEEDMSPRDNFVIDDEEEEEEEEEEEEEEEEEEEEE |
| SLKYIYMYLTPSDSFNLVRRNRNLDEEDMSPRDNFVMDDEEEEEEEEEEEEEEEEEEEEE |
| TDQLKPTESGPKVKKCEVKVNEPLIKVKIICPLKGSVEKLYDNIEYVPKKSPYVVLTKEE |
| TDQLKPTESSPKVKKCEVKVNEPLIKVKIICPLKGSVEKLYDNIEYVPKKSPYVVLTKEE |
| TKLKEKLLSKLIYGLLISPTVNEKENNFKEGVIEFTLPPVVHKATVFYFICDNSKTEDDN |
| TKLKENLLSKLIYGLLISPTVNEKENNFKE |
| VDEYYEDQDGDTYDSTIKNEDVDEEVGEEVGEEVGEEVGEEVGEEVGEEVGEEVGEEVGE |
| VDTGPVLDNSTFEKYFKNIKIKPDKFFEKVINENDDTEEEKDLESILPGAIVSPMKVLKK |
| VDTGPVLDNSTFEKYFKNIKIKPDKFFEKVINEYDDTEEEKDLESILPGAIVSPMKVLKK |
| VEEGVQNEEYKKFSLKPSLVFDDNNNDIKVIGKEKNEVSISLALKGVYGNRIFTFDKNGK |
| VKGCSFDNEHAHMFSYNKTNVKNCIIDAKPKDLIGFVCPSGTLKLTNCFKDAIVHTNLTN |

**Table S9A**: Changes in amino acid letter are coloured

| Antigen |  | Peptide | Antigenicity | Protein accession number | Position of the peptide |
| --- | --- | --- | --- | --- | --- |
| Pfs25 | P1 | ILDTSNPVKTAVCSCNIGKVPNVQNQNKCSKDGETKCSLKCLKENETCKAVDGIYKCDCK | 0.7589 | UIT08906.1 | 120-180 |
|  | P2 | DGFIIDNESSICTAFSAYNILNLSIMFILFSVCFFIM | 0.5775 | AAD39544.1 | 180-217 |
| Pfs28 | P3 | MNTYFKVLLFLFIQLYITLNKARVTENTICKYGYLIQMSNHYECKCIEGYVLINEDTCGK | 0.9098 | AAT00624.1 | 1-60 |
|  | P4 | SNGGGNTVDQADTSYSVINGVTLTHVLIVCSIFIKLLI | 0.5793 | AAT00624.1 | 180-218 |
| Pfs48/45 | P5 | SSNDSSKHTFTDSLDISLVDDNAHISCNVHLSEPKYNHLVGLNCPGDIVPDCFFLVYQPE | 1.0393 | AAL74377.1 | 300-360 |
|  | P6 | SSNDSSKHTFTDSLDISLVDDNAHISCNVHLSEPKYNHLVGLNCPGDIIPDCFFQVYQPE | 1.0172 | ABO41490.1 | 293-353 |
|  | P7 | MMLYISAKKAQVAFILYIVLVLRIISGNNDFYKPSSLNSEISGFIGYKCNFSNEGVHNLK | 0.6462 | AAL74379.1 | 1-60 |
|  | P8 | MMLYISAKKAQVAFILYIVLVLRIISGNNDFYNPSALNSEISGFIGYKCNFSNEGVHNLK | 0.6224 | AAL74380.1 | 1-60 |
|  | P9 | MMLYISAKKAQVAFILYIVLVLRIISGNNDFCKPSSLNSEISGFIGYKCNFSNEGVHDLK | 0.6155 | AAL74350.1 | 1-60 |
| Pfs230 | P10 | TKLKENLLSKLIYGLLISPTVNEKENNFKE | 1.1821 | UIT08906.1 | 124-154 |
|  | P11 | EEEEYDDYVYEESGDETEEQLQEEHQEEVGAESSEESFNDEDEDSVEARDGDMIR | 0.9218 | AAA29734.1 | 300-355 |
|  | P12 | VDTGPVLDNSTFEKYFKNIKIKPDKFFEKVINENDDTEEEKDLESILPGAIVSPMKVLKK | 0.9154 | AAG12332.1 | 1800-1860 |
|  | P13 | VEEGVQNEEYKKFSLKPSLVFDDNNNDIKVIGKEKNEVSISLALKGVYGNRIFTFDKNGK | 0.9056 | AAA29734.1 | 2000-2060 |
|  | P14 | EEEEEYDDYVYEESGDETEEQLQEEHQEEVGAESSEESFNDEDEDSVEARDGDMIRVDEY | 0.8761 | AAG12332.1 | 300-360 |

**Table S9B**: Similarity search of the peptide’s relationship with other *Plasmodium* species. The ones with best E-value are downloaded.

| peptide | Scientific Name | Query Cover | E value | Per. ident | Accession |
| --- | --- | --- | --- | --- | --- |
| P1 | Plasmodium malariae | 100% | 3.00E-21 | 70.49 | [XP_028862196.1](https://www.ncbi.nlm.nih.gov/protein/XP_028862196.1?report=genbank&log$=prottop&blast_rank=1&RID=GSMV06VA013) |
|  | Plasmodium knowlesi | 98% | 3.00E-17 | 61.02 | [AND94994.1](https://www.ncbi.nlm.nih.gov/protein/AND94994.1?report=genbank&log$=prottop&blast_rank=2&RID=GSMV06VA013) |
|  | Plasmodium ovale | 100% | 2.00E-15 | 55.00 | [AND95000.1](https://www.ncbi.nlm.nih.gov/protein/AND95000.1?report=genbank&log$=prottop&blast_rank=3&RID=GSMV06VA013) |
|  | Plasmodium vivax | 98% | 6.00E-15 | 55.93 | [AAV33641.1](https://www.ncbi.nlm.nih.gov/protein/AAV33641.1?report=genbank&log$=prottop&blast_rank=4&RID=GSMV06VA013) |
| P2 | Plasmodium malariae | 100% | 1.00E-05 | 51.35 | [SBS98341.1](https://www.ncbi.nlm.nih.gov/protein/SBS98341.1?report=genbank&log$=prottop&blast_rank=1&RID=GSN7WSHD013) |
|  | Plasmodium ovale curtisi | 100% | 4.00E-05 | 45.95 | [SBS84251.1](https://www.ncbi.nlm.nih.gov/protein/SBS84251.1?report=genbank&log$=prottop&blast_rank=2&RID=GSN7WSHD013) |
| P3 | Plasmodium malariae | 96% | 7.00E-18 | 56.9 | [XP_028862196.1](https://www.ncbi.nlm.nih.gov/protein/XP_028862196.1?report=genbank&log$=prottop&blast_rank=1&RID=GSNE40EE016) |
|  | Plasmodium ovale | 96% | 3.00E-15 | 55.17 | [BAB43948.1](https://www.ncbi.nlm.nih.gov/protein/BAB43948.1?report=genbank&log$=prottop&blast_rank=2&RID=GSNE40EE016) |
|  | Plasmodium vivax | 96% | 3.00E-15 | 53.45 | [AND95026.1](https://www.ncbi.nlm.nih.gov/protein/AND95026.1?report=genbank&log$=prottop&blast_rank=3&RID=GSNE40EE016) |
|  | Plasmodium knowlesi strain H | 100% | 2.00E-13 | 46.67 | [XP_002261828.1](https://www.ncbi.nlm.nih.gov/protein/XP_002261828.1?report=genbank&log$=prottop&blast_rank=4&RID=GSNE40EE016) |
| P4 |  |  |  |  |  |
| P5 | Plasmodium malariae | 98% | 7.00E-23 | 62.71 | [AUR33952.1](https://www.ncbi.nlm.nih.gov/protein/AUR33952.1?report=genbank&log$=prottop&blast_rank=1&RID=GSNRSX2V013) |
|  | Plasmodium ovale curtisi | 88% | 3.00E-18 | 56.6 | [SBS85830.1](https://www.ncbi.nlm.nih.gov/protein/SBS85830.1?report=genbank&log$=prottop&blast_rank=2&RID=GSNRSX2V013) |
|  | Plasmodium knowlesi strain H | 95% | 1.00E-16 | 50.88 | [XP_002259885.1](https://www.ncbi.nlm.nih.gov/protein/XP_002259885.1?report=genbank&log$=prottop&blast_rank=3&RID=GSNRSX2V013) |
|  | Plasmodium vivax | 98% | 2.00E-16 | 47.46 | [AFB76627.1](https://www.ncbi.nlm.nih.gov/protein/AFB76627.1?report=genbank&log$=prottop&blast_rank=4&RID=GSNRSX2V013) |
| P6 | Plasmodium malariae | 98% | 9.00E-24 | 62.71 | [AUR33952.1](https://www.ncbi.nlm.nih.gov/protein/AUR33952.1?report=genbank&log$=prottop&blast_rank=1&RID=GSP2U4JN016) |
|  | Plasmodium ovale curtisi | 88% | 3.00E-19 | 58.49 | [SBS85830.1](https://www.ncbi.nlm.nih.gov/protein/SBS85830.1?report=genbank&log$=prottop&blast_rank=2&RID=GSP2U4JN016) |
|  | Plasmodium knowlesi strain H | 95% | 8.00E-18 | 52.63 | [XP_002259885.1](https://www.ncbi.nlm.nih.gov/protein/XP_002259885.1?report=genbank&log$=prottop&blast_rank=3&RID=GSP2U4JN016) |
|  | Plasmodium vivax | 98% | 1.00E-17 | 49.15 | [AMQ67085.1](https://www.ncbi.nlm.nih.gov/protein/AMQ67085.1?report=genbank&log$=prottop&blast_rank=4&RID=GSP2U4JN016) |
| P7 | Plasmodium ovale curtisi | 98% | 2.00E-08 | 32.2 | [SBS85830.1](https://www.ncbi.nlm.nih.gov/protein/SBS85830.1?report=genbank&log$=prottop&blast_rank=1&RID=GSPC589Z013) |
|  | Plasmodium knowlesi strain H | 65% | 5.00E-08 | 48.72 | [XP_002259885.1](https://www.ncbi.nlm.nih.gov/protein/XP_002259885.1?report=genbank&log$=prottop&blast_rank=2&RID=GSPC589Z013) |
|  | Plasmodium vivax | 63% | 7.00E-07 | 47.37 | [ALS19525.1](https://www.ncbi.nlm.nih.gov/protein/ALS19525.1?report=genbank&log$=prottop&blast_rank=3&RID=GSPC589Z013) |
|  | Plasmodium malariae | 56% | 1.00E-05 | 44.12 | [SBT79956.1](https://www.ncbi.nlm.nih.gov/protein/SBT79956.1?report=genbank&log$=prottop&blast_rank=4&RID=GSPC589Z013) |
| P8 | Plasmodium knowlesi strain H | 65% | 4.00E-08 | 48.72 | [XP_002259885.1](https://www.ncbi.nlm.nih.gov/protein/XP_002259885.1?report=genbank&log$=prottop&blast_rank=1&RID=GSK2VD1U013) |
|  | Plasmodium vivax | 63% | 9.00E-07 | 47.37 | [ALS19525.1](https://www.ncbi.nlm.nih.gov/protein/ALS19525.1?report=genbank&log$=prottop&blast_rank=2&RID=GSK2VD1U013) |
|  | Plasmodium malariae | 56% | 1.00E-05 | 44.12 | [SBT79956.1](https://www.ncbi.nlm.nih.gov/protein/SBT79956.1?report=genbank&log$=prottop&blast_rank=3&RID=GSK2VD1U013) |
| P9 | Plasmodium knowlesi strain H | 65% | 2.00E-07 | 46.15 | [XP_002259885.1](https://www.ncbi.nlm.nih.gov/protein/XP_002259885.1?report=genbank&log$=prottop&blast_rank=1&RID=GSKD9HVR016) |
|  | Plasmodium vivax | 63% | 3.00E-06 | 44.74 | [ALS19464.1](https://www.ncbi.nlm.nih.gov/protein/ALS19464.1?report=genbank&log$=prottop&blast_rank=2&RID=GSKD9HVR016) |
|  | Plasmodium malariae | 55% | 6.00E-05 | 42.42 | [SBT79956.1](https://www.ncbi.nlm.nih.gov/protein/SBT79956.1?report=genbank&log$=prottop&blast_rank=3&RID=GSKD9HVR016) |
| P10 | Plasmodium vivax | 86% | 4.00E-06 | 65.38 | [UYP39350.1](https://www.ncbi.nlm.nih.gov/protein/UYP39350.1?report=genbank&log$=prottop&blast_rank=1&RID=GSKNDV3N013) |
| P11 |  |  |  |  |  |
| P12 | Plasmodium malariae | 100% | 2.00E-11 | 53.33 | [SBT70600.1](https://www.ncbi.nlm.nih.gov/protein/SBT70600.1?report=genbank&log$=prottop&blast_rank=1&RID=GSM2FTSW016) |
|  | Plasmodium knowlesi strain H | 80% | 1.00E-09 | 56.25 | [XP_002257945.2](https://www.ncbi.nlm.nih.gov/protein/XP_002257945.2?report=genbank&log$=prottop&blast_rank=2&RID=GSM2FTSW016) |
| P13 | Plasmodium knowlesi strain H | 88% | 2.00E-13 | 62.26 | [XP_002257945.2](https://www.ncbi.nlm.nih.gov/protein/XP_002257945.2?report=genbank&log$=prottop&blast_rank=1&RID=GSM9Y2PM016) |
|  | Plasmodium malariae | 96% | 2.00E-13 | 58.62 | [SBT70600.1](https://www.ncbi.nlm.nih.gov/protein/SBT70600.1?report=genbank&log$=prottop&blast_rank=2&RID=GSM9Y2PM016) |
|  | Plasmodium vivax | 86% | 2.00E-09 | 50 | [BAL44502.1](https://www.ncbi.nlm.nih.gov/protein/BAL44502.1?report=genbank&log$=prottop&blast_rank=3&RID=GSM9Y2PM016) |
| P14 |  |  |  |  |  |

**Table S9C**: Similarity search of the fused antigen’s relationship with other *Plasmodium* species. The ones with best E-value are downloaded.

|  | *Plasmodium* species | Description | Scientific Name | E value | Per. ident | Accession |
| --- | --- | --- | --- | --- | --- | --- |
| FAVC-FSE | *P. vivax* | ookinete surface protein Pvs25 [Plasmodium vivax] | Plasmodium vivax | 2.00E-13 | 52.24 | [AAV33641.1](https://www.ncbi.nlm.nih.gov/protein/AAV33641.1?report=genbank&log$=prottop&blast_rank=7&RID=GVYBZHZA013) |
|  |  | transmission blocking target antigen precursor (Pvs48/45) [Plasmodium vivax] | Plasmodium vivax | 4.00E-13 | 40.26 | [BAQ02887.1](https://www.ncbi.nlm.nih.gov/protein/BAQ02887.1?report=genbank&log$=prottop&blast_rank=10&RID=GVYBZHZA013) |
|  |  | ookinete surface protein pvs28 [Plasmodium vivax] | Plasmodium vivax | 3.00E-12 | 45.95 | [ACJ54133.1](https://www.ncbi.nlm.nih.gov/protein/ACJ54133.1?report=genbank&log$=prottop&blast_rank=12&RID=GVYBZHZA013) |
|  | *P. ovale* | 6-cysteine protein (P48/45) [Plasmodium ovale curtisi] | Plasmodium ovale curtisi | 3.00E-15 | 47.89 | [SBS85830.1](https://www.ncbi.nlm.nih.gov/protein/SBS85830.1?report=genbank&log$=prottop&blast_rank=6&RID=GVYBZHZA013) |
|  |  | ookinete surface protein Pos25 [Plasmodium ovale] | Plasmodium ovale | 2.00E-13 | 53.23 | [BAB43948.1](https://www.ncbi.nlm.nih.gov/protein/BAB43948.1?report=genbank&log$=prottop&blast_rank=8&RID=GVYBZHZA013) |
|  |  | 28 kDa ookinete surface protein, putative [Plasmodium ovale] | Plasmodium ovale | 3.00E-13 | 51.39 | [SCP03940.1](https://www.ncbi.nlm.nih.gov/protein/SCP03940.1?report=genbank&log$=prottop&blast_rank=9&RID=GVYBZHZA013) |
|  | *P. malariae* | 25 kDa ookinete surface antigen precursor (P25) [Plasmodium malariae] | Plasmodium malariae | 4.00E-19 | 68.25 | [SBS98341.1](https://www.ncbi.nlm.nih.gov/protein/SBS98341.1?report=genbank&log$=prottop&blast_rank=4&RID=GVYBZHZA013) |
|  |  | 28 kDa ookinete surface protein, putative [Plasmodium malariae] | Plasmodium malariae | 3.00E-10 | 47.69 | [SBT71852.1](https://www.ncbi.nlm.nih.gov/protein/SBT71852.1?report=genbank&log$=prottop&blast_rank=15&RID=GVYBZHZA013) |
|  |  | 6-cysteine protein (P230) [Plasmodium malariae] | Plasmodium malariae | 0.001 | 51.16 | [SBS91837.1](https://www.ncbi.nlm.nih.gov/protein/SBS91837.1?report=genbank&log$=prottop&blast_rank=17&RID=GVYBZHZA013) |
|  | *P. knowlesi* | ookinete surface protein P25, putative [Plasmodium knowlesi strain H] | Plasmodium knowlesi strain H | 5.00E-16 | 49.44 | [XP_002261828.1](https://www.ncbi.nlm.nih.gov/protein/XP_002261828.1?report=genbank&log$=prottop&blast_rank=5&RID=GVYBZHZA013) |
|  |  | ookinete surface protein P28, putative [Plasmodium knowlesi strain H] | Plasmodium knowlesi strain H | 2.00E-09 | 44.44 | [XP_002261827.1](https://www.ncbi.nlm.nih.gov/protein/XP_002261827.1?report=genbank&log$=prottop&blast_rank=16&RID=GVYBZHZA013) |
| FAVC-CTB | *P. vivax* | ookinete surface protein Pvs25 [Plasmodium vivax] | Plasmodium vivax | 3.00E-14 | 50.72 | [AAV33641.1](https://www.ncbi.nlm.nih.gov/protein/AAV33641.1?report=genbank&log$=prottop&blast_rank=9&RID=GVYARHZE013) |
|  |  | gamete surface antigen Pvs48/45 [Plasmodium vivax] | Plasmodium vivax | 3.00E-13 | 47.46 | [AFB76625.1](https://www.ncbi.nlm.nih.gov/protein/AFB76625.1?report=genbank&log$=prottop&blast_rank=12&RID=GVYARHZE013) |
|  |  | sexual stage surface protein Pvs28 [Plasmodium vivax] | Plasmodium vivax | 2.00E-12 | 55.17 | [AFE88196.1](https://www.ncbi.nlm.nih.gov/protein/AFE88196.1?report=genbank&log$=prottop&blast_rank=14&RID=GVYARHZE013) |
|  | *P. ovale* | 6-cysteine protein (P48/45) [Plasmodium ovale curtisi] | Plasmodium ovale curtisi | 8.00E-16 | 55.36 | [SBS85830.1](https://www.ncbi.nlm.nih.gov/protein/SBS85830.1?report=genbank&log$=prottop&blast_rank=7&RID=GVYARHZE013) |
|  |  | 25 kDa ookinete surface antigen precursor (P25) [Plasmodium ovale curtisi] | Plasmodium ovale curtisi | 9.00E-15 | 45.88 | [SBS84251.1](https://www.ncbi.nlm.nih.gov/protein/SBS84251.1?report=genbank&log$=prottop&blast_rank=8&RID=GVYARHZE013) |
|  |  | 28 kDa ookinete surface protein (P28) [Plasmodium ovale curtisi] | Plasmodium ovale curtisi | 1.00E-13 | 51.39 | [SBS84253.1](https://www.ncbi.nlm.nih.gov/protein/SBS84253.1?report=genbank&log$=prottop&blast_rank=10&RID=GVYARHZE013) |
|  | *P. malariae* | gametocyte antigen p48/45 [Plasmodium malariae] | Plasmodium malariae | 3.00E-21 | 62.3 | [AUR33952.1](https://www.ncbi.nlm.nih.gov/protein/AUR33952.1?report=genbank&log$=prottop&blast_rank=4&RID=GVYARHZE013) |
|  |  | 25 kDa ookinete surface antigen precursor (P25) [Plasmodium malariae] | Plasmodium malariae | 1.00E-19 | 68.25 | [SBS98341.1](https://www.ncbi.nlm.nih.gov/protein/SBS98341.1?report=genbank&log$=prottop&blast_rank=5&RID=GVYARHZE013) |
|  |  | 28 kDa ookinete surface protein, putative [Plasmodium malariae] | Plasmodium malariae | 1.00E-10 | 47.69 | [SBT71852.1](https://www.ncbi.nlm.nih.gov/protein/SBT71852.1?report=genbank&log$=prottop&blast_rank=15&RID=GVYARHZE013) |
|  |  | 6-cysteine protein (P230) [Plasmodium malariae] | Plasmodium malariae | 4.00E-04 | 51.16 | [SBS91837.1](https://www.ncbi.nlm.nih.gov/protein/SBS91837.1?report=genbank&log$=prottop&blast_rank=18&RID=GVYARHZE013) |
|  | *P. knowlesi* | ookinete surface protein P25, putative [Plasmodium knowlesi strain H] | Plasmodium knowlesi strain H | 2.00E-16 | 49.44 | [XP_002261828.1](https://www.ncbi.nlm.nih.gov/protein/XP_002261828.1?report=genbank&log$=prottop&blast_rank=6&RID=GVYARHZE013) |
|  |  | ookinete surface protein P28, putative [Plasmodium knowlesi strain H] | Plasmodium knowlesi strain H | 6.00E-10 | 44.44 | [XP_002261827.1](https://www.ncbi.nlm.nih.gov/protein/XP_002261827.1?report=genbank&log$=prottop&blast_rank=17&RID=GVYARHZE013) |


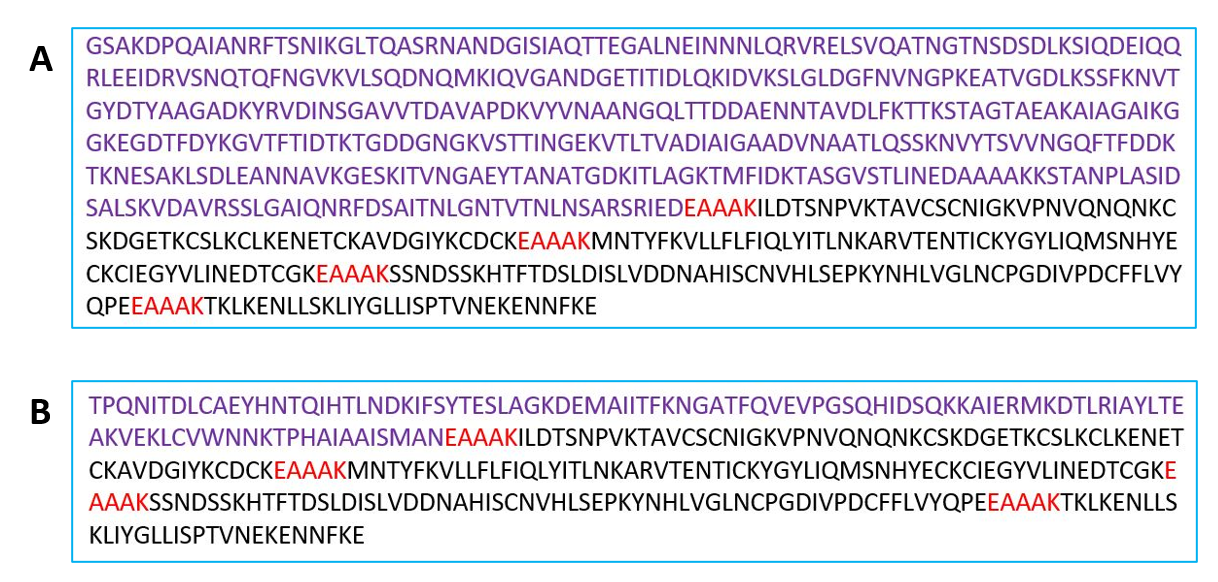


**Figure S1**: The sequences for FAVC. **A**. FAVC-FSE. **B**. FAVC-CTB. The purple letters represent the amino acids for adjuvants (Flagellin *Salmonella enterica* (FSE) and Cholera Toxin B (CTB)), red letters represent the linkers and the black letters represent the antigenic peptides

**Table S10**: Comparison of models for physical properties of fused antigen vaccine candidate (FAVC)

|  | ProtParam tool | | ThermoFisher peptide analyzing tool | | Prot pi protein/peptide calculator | | Biosynth peptide calculator | |
| --- | --- | --- | --- | --- | --- | --- | --- | --- |
|  | FAVC-FSE | FAVC-CTB | FAVC-FSE | FAVC-CTB | FAVC-FSE | FAVC-CTB | FAVC-FSE | FAVC-CTB |
| GRAVY | -0.39 | -0.31 | -0.39 | -0.31 |  |  | -0.39 | -0.31 |
| Isoelectric point (PI) | 5.19 | 6.31 | 5.0 | 6.30 | 5.21 | 6.37 | 5.20 | 6.32 |
| Net charge at pH 7.4 |  |  |  |  | -15.19 | -5.42 | -14.43 | -4.48 |

**Table S11**: Secondary structure composition of FAVCs with different modelling tools

|  | SOPMA | | GOR4 | | PROTEUS2 | |
| --- | --- | --- | --- | --- | --- | --- |
|  | FAVC-FSE | FAVC-CTB | FAVC-FSE | FAVC-CTB | FAVC-FSE | FAVC-CTB |
| Alpha helix | 37.25 | 42.34 | 30.84 | 27.93 | 28.00 | 23.00 |
| Extended strand | 23.82 | 19.22 | 20.61 | 21.02 |  |  |
| Beta turn/sheet | 9.00 | 7.21 |  |  | 23.00 | 32.00 |
| Random coil | 29.92 | 31.23 | 48.55 | 51.05 | 50.00 | 45.00 |

**Table S12**: Ramachandran plot analysis (%) with the initial generated 3D structures from different models

|  | SWISS | | PROCHEK | |
| --- | --- | --- | --- | --- |
|  | FAVC-FSE | FAVC-CTB | FAVC-FSE | FAVC-CTB |
| Robetta | 88.82 | 88.52 | 79.30 | 78.60 |
| trRosetta | 95.25 | 90.94 | 90.50 | 87.40 |
| iDRUG | 83.92 | 82.18 | 76.00 | 74.80 |

**Table S13**: CD8^+^ epitopes with their HLA alleles and antigenicity score for P1, P3, P5, P10, FSE, and CTB

|  | Peptide | Allele | Antigenicity score |
| --- | --- | --- | --- |
| P1 | SKDGETKCS | HLA-C*12:03,HLA-C*05:01 | 2.7283 |
|  | CSKDGETKC | HLA-C*12:03,HLA-C*03:03 | 2.4277 |
|  | KCSKDGETK | HLA-C*12:03,HLA-C*03:03 | 2.409 |
|  | QNKCSKDGE | HLA-C*12:03,HLA-C*03:03 | 1.9355 |
|  | NQNKCSKDG | HLA-C*12:03,HLA-C*07:01,HLA-C*05:01,HLA-C*03:03 | 1.928 |
|  | KDGETKCSL | HLA-C*12:03,HLA-C*14:02 | 1.9027 |
|  | GETKCSLKC | HLA-C*12:03,HLA-C*03:03 | 1.8916 |
| P3 | YFKVLLFLF | HLA-A*23:01,HLA-C*12:03,HLA-A*24:02,HLA-A*29:02,HLA-B*15:02,HLA-C*14:02 | 2.6377 |
|  | FKVLLFLFI | HLA-C*03:03,HLA-C*12:03,HLA-A*02:06,HLA-B*15:02 | 2.5524 |
|  | LFLFIQLYI | HLA-C*14:02,HLA-C*03:03,HLA-C*12:03,HLA-A*23:01 | 2.5186 |
|  | NTYFKVLLF | HLA-C*03:03,HLA-C*14:02,HLA-C*12:03,HLA-A*32:01,HLA-A*29:02,HLA-C*07:01 | 2.3689 |
|  | TYFKVLLFL | HLA-C*14:02,HLA-C*12:03,HLA-A*23:01,HLA-B*15:02 | 2.1777 |
|  | LFIQLYITL | HLA-C*03:03,HLA-C*14:02,HLA-C*12:03,HLA-B*15:02 | 1.9928 |
|  | LLFLFIQLY | HLA-C*12:03,HLA-A*29:02,HLA-B*15:01,HLA-B*15:02,HLA-C*07:01,HLA-C*03:03,HLA-C*14:02,HLA-A*03:01 | 1.9535 |
| P5 | GDIVPDCFF | HLA-C*03:03,HLA-C*12:03,HLA-B*15:02 | 2.8623 |
|  | PGDIVPDCF | HLA-C*05:01,HLA-C*12:03,HLA-C*07:02 | 2.7054 |
|  | NAHISCNVH | HLA-C*03:03,HLA-C*12:03,HLA-C*14:02,HLA-C*15:02 | 2.2105 |
|  | CPGDIVPDC | HLA-C*12:03,HLA-C*03:03,HLA-B*53:01 | 2.0034 |
|  | DIVPDCFFL | HLA-C*03:03,HLA-C*12:03,HLA-C*07:02,HLA-B*15:02 | 1.9996 |
|  | DDNAHISCN | HLA-C*12:03,HLA-C*07:01,HLA-C*03:03 | 1.8599 |
|  | AHISCNVHL | HLA-C*03:03,HLA-C*12:03,HLA-B*39:01 | 1.8435 |
| P10 | SKLIYGLLI | HLA-C*12:03 | 1.9106 |
|  | LIYGLLISP | HLA-C*12:03,HLA-C*14:02,HLA-C*03:03,HLA-A*02:06 | 1.6789 |
|  | TKLKENLLS | HLA-C*12:03,HLA-C*03:03 | 1.4642 |
|  | KLIYGLLIS | HLA-C*12:03,HLA-C*14:02 | 1.428 |
|  | NEKENNFKE | HLA-C*12:03 | 1.363 |
|  | LSKLIYGLL | HLA-C*12:03,HLA-C*03:03,HLA-B*15:02 | 1.2205 |
|  | NLLSKLIYG | HLA-C*14:02,HLA-C*03:03,HLA-C*12:03 | 1.2149 |
| FSE adjuvant | TKTGDDGNG | HLA-C*03:03,HLA-C*12:03 | 2.807 |
|  | IKGGKEGDT | HLA-C*12:03,HLA-C*03:03 | 2.7496 |
|  | DTKTGDDGN | HLA-C*12:03,HLA-C*03:03 | 2.694 |
|  | AIKGGKEGD | HLA-C*12:03,HLA-C*03:03 | 2.5329 |
|  | TNGTNSDSD | HLA-C*12:03,HLA-C*03:03,HLA-C*14:02 | 2.3579 |
|  | GDDGNGKVS | HLA-C*12:03,HLA-C*05:01,HLA-C*08:02,HLA-C*14:02 | 2.3053 |
|  | DVKSLGLDG | HLA-C*12:03 | 2.2955 |
| CTB adjuvant | TFQVEVPGS | HLA-C*14:02,HLA-C*12:03,HLA-C*03:03,HLA-C*07:02 | 2.3897 |
|  | FQVEVPGSQ | HLA-C*12:03,HLA-C*03:03,HLA-A*02:06 | 2.2985 |
|  | ATFQVEVPG | HLA-C*12:03,HLA-C*03:03,HLA-C*14:02 | 2.2938 |
|  | GATFQVEVP | HLA-C*03:03,HLA-C*12:03,HLA-C*15:02,HLA-C*14:02 | 1.4356 |
|  | QVEVPGSQH | HLA-C*05:01,HLA-C*12:03,HLA-C*03:03 | 1.3731 |
|  | LNDKIFSYT | HLA-C*12:03,HLA-C*05:01,HLA-C*14:02 | 1.3338 |
|  | ERMKDTLRI | HLA-C*12:03,HLA-C*07:01,HLA-C*14:02,HLA-C*06:02 | 1.2089 |

**Table S14**: CD4^+^ epitopes with their HLA alleles and antigenicity score for P1, P3, P5, P10, FSE, and CTB

|  | Peptide | Allele | Antigenicity score |
| --- | --- | --- | --- |
| P1 | DTSNPVKTAVCSCNI | HLA-DRB1*07:01 | 0.8015 |
|  | ENETCKAVDGIYKCD | HLA-DRB5*01:01 | 0.7392 |
|  | KENETCKAVDGIYKC | HLA-DRB5*01:01 | 0.6237 |
|  | TSNPVKTAVCSCNIG | HLA-DRB1*07:01 | 0.6057 |
|  | ETCKAVDGIYKCDCK | HLA-DRB5*01:01 | 0.5663 |
| P3 | TYFKVLLFLFIQLYI | HLA-DPA1*01:03/DPB1*02:01 | 2.1585 |
|  | YFKVLLFLFIQLYIT | HLA-DPA1*01:03/DPB1*02:01,HLA-DPA1*02:01/DPB1*01:01 | 2.1055 |
|  | FKVLLFLFIQLYITL | HLA-DRB1*01:01 | 2.0201 |
|  | NTYFKVLLFLFIQLY | HLA-DPA1*01:03/DPB1*02:01,HLA-DPA1*02:01/DPB1*01:01 | 1.9872 |
|  | FKVLLFLFIQLYITL | HLA-DPA1*01:03/DPB1*02:01,HLA-DPA1*02:01/DPB1*01:01 | 1.9736 |
|  | MNTYFKVLLFLFIQL | HLA-DPA1*01:03/DPB1*02:01,HLA-DPA1*02:01/DPB1*01:01 | 1.817 |
| P5 | GLNCPGDIVPDCFFL | HLA-DQA1*05:01/DQB1*02:01 | 2.0727 |
|  | VGLNCPGDIVPDCFF | HLA-DQA1*05:01/DQB1*02:01 | 2.0698 |
|  | LNCPGDIVPDCFFLV | HLA-DPA1*01:03/DPB1*02:01,HLA-DRB3*01:01,HLA-DQA1*05:01/DQB1*02:01 | 1.8761 |
|  | LVGLNCPGDIVPDCF | HLA-DRB1*13:02,HLA-DQA1*05:01/DQB1*02:01 | 1.8341 |
|  | NCPGDIVPDCFFLVY | HLA-DQA1*05:01/DQB1*02:01 | 1.8334 |
|  | PGDIVPDCFFLVYQP | HLA-DPA1*01:03/DPB1*02:01,HLA-DRB3*01:01,HLA-DPA1*01:03/DPB1*04:01 | 1.7606 |
|  | NCPGDIVPDCFFLVY | HLA-DRB3*01:01 | 1.7287 |
| P10 | LKENLLSKLIYGLLI | HLA-DRB1*12:01,HLA-DPA1*03:01/DPB1*04:02,HLA-DRB1*01:01,HLA-DPA1*01:03/DPB1*04:01,HLA-DPA1*02:01/DPB1*01:01,HLA-DPA1*01:03/DPB1*02:01,HLA-DRB1*15:01 | 1.6029 |
|  | KENLLSKLIYGLLIS | HLA-DPA1*03:01/DPB1*04:02,HLA-DRB1*12:01,HLA-DRB1*01:01,HLA-DPA1*01:03/DPB1*04:01,HLA-DPA1*02:01/DPB1*01:01,HLA-DPA1*01:03/DPB1*02:01,HLA-DRB1*15:01 | 1.4532 |
|  | ENLLSKLIYGLLISP | HLA-DPA1*03:01/DPB1*04:02,HLA-DRB1*12:01,HLA-DPA1*01:03/DPB1*04:01,HLA-DPA1*02:01/DPB1*01:01,HLA-DRB1*01:01,HLA-DPA1*01:03/DPB1*02:01 | 1.3341 |
|  | KLKENLLSKLIYGLL | HLA-DRB1*12:01,HLA-DPA1*03:01/DPB1*04:02,HLA-DRB1*01:01,HLA-DPA1*01:03/DPB1*04:01,HLA-DPA1*02:01/DPB1*01:01,HLA-DRB1*13:02 | 1.273 |
|  | LLSKLIYGLLISPTV | HLA-DRB1*01:01,HLA-DRB1*09:01,HLA-DPA1*03:01/DPB1*04:02,HLA-DRB1*12:01,HLA-DRB1*04:05,HLA-DPA1*02:01/DPB1*01:01,HLA-DRB1*04:01,HLA-DPA1*01:03/DPB1*04:01,HLA-DRB1*15:01,HLA-DPA1*01:03/DPB1*02:01,HLA-DRB4*01:01 | 1.1842 |
|  | TKLKENLLSKLIYGL | HLA-DRB1*12:01,HLA-DPA1*03:01/DPB1*04:02,HLA-DRB1*01:01,HLA-DRB1*13:02,HLA-DPA1*02:01/DPB1*01:01,HLA-DPA1*01:03/DPB1*04:01 | 1.1451 |
|  | LSKLIYGLLISPTVN | HLA-DRB1*01:01,HLA-DRB1*09:01,HLA-DRB1*04:05,HLA-DPA1*03:01/DPB1*04:02,HLA-DRB1*04:01,HLA-DRB1*15:01,HLA-DRB1*12:01,HLA-DPA1*02:01/DPB1*01:01,HLA-DRB4*01:01,HLA-DRB5*01:01 | 1.1016 |
| FSE adjuvant | GDDGNGKVSTTINGE | HLA-DQA1*05:01/DQB1*03:01 | 2.4132 |
|  | TGDDGNGKVSTTING | HLA-DQA1*05:01/DQB1*03:01 | 2.0716 |
|  | KAIAGAIKGGKEGDT | HLA-DQA1*05:01/DQB1*03:01 | 1.9219 |
|  | AKAIAGAIKGGKEGD | HLA-DQA1*05:01/DQB1*03:01,HLA-DRB5*01:01 | 1.8771 |
|  | TKTGDDGNGKVSTTI | HLA-DQA1*05:01/DQB1*03:01 | 1.8599 |
|  | NGKVSTTINGEKVTL | HLA-DRB1*01:01 | 1.6593 |
|  | KTGDDGNGKVSTTIN | HLA-DQA1*05:01/DQB1*03:01 | 1.6305 |
| CTB adjuvant | GATFQVEVPGSQHID | HLA-DRB3*01:01 | 1.1206 |
|  | ERMKDTLRIAYLTEA | HLA-DPA1*03:01/DPB1*04:02,HLA-DRB1*11:01,HLA-DRB1*01:01,HLA-DRB4*01:01,HLA-DRB4*01:01,HLA-DRB1*01:01,HLA-DPA1*03:01/DPB1*04:02,HLA-DPA1*02:01/DPB1*01:01 | 0.9987 |
|  | FKNGATFQVEVPGSQ | HLA-DQA1*05:01/DQB1*03:01,HLA-DRB1*09:01,HLA-DRB1*07:01,HLA-DRB1*04:01 | 0.8779 |
|  | LNDKIFSYTESLAGK | HLA-DRB1*07:01,HLA-DRB1*04:01,HLA-DRB1*15:01,HLA-DPA1*01:03/DPB1*02:01,HLA-DRB1*09:01,HLA-DRB1*04:05,HLA-DRB1*01:01,HLA-DRB1*08:02,HLA-DPA1*03:01/DPB1*04:02 | 0.8615 |
|  | TFKNGATFQVEVPGS | HLA-DRB1*09:01,HLA-DRB1*07:01,HLA-DRB1*04:01,HLA-DQA1*05:01/DQB1*03:01,HLA-DRB1*01:01 | 0.7831 |
|  | VEVPGSQHIDSQKKA | HLA-DRB5*01:01 | 0.7431 |
|  | KDTLRIAYLTEAKVE | HLA-DRB1*01:01,HLA-DRB4*01:01,HLA-DPA1*03:01/DPB1*04:02,HLA-DRB1*09:01,HLA-DRB1*12:01,HLA-DPA1*02:01/DPB1*01:01,HLA-DRB1*15:01 | 0.7325 |

**Table S15**: Detailed population coverage for countries of combined class I and II

| **Area** | **coverage** | **Area** | **coverage** | **Area** | **coverage** | **Area** | **coverage** |
| --- | --- | --- | --- | --- | --- | --- | --- |
| Ireland Northern | 98.69% | Italy | 92.48% | India Asian | 87.84% | Israel | 78.59% |
| Ireland Northern Caucasoid | 98.69% | Italy Caucasoid | 92.48% | Australia | 87.82% | East Africa | 78.47% |
| England Caucasoid | 98.57% | Morocco Caucasoid | 92.26% | Singapore | 87.81% | United Kingdom | 78.19% |
| Ireland South | 98.48% | Brazil Mixed | 92.14% | Thailand | 87.70% | United Kingdom Caucasoid | 78.19% |
| Ireland South Caucasoid | 98.48% | Spain Caucasoid | 92.14% | Thailand Oriental | 87.70% | Sao Tome and Principe | 77.33% |
| Germany | 98.02% | Spain | 92.04% | West Africa | 86.37% | Sao Tome and Principe Black | 77.33% |
| Germany Caucasoid | 98.02% | Taiwan | 91.84% | Iran Persian | 86.33% | Ecuador | 77.16% |
| Russia Other | 98.00% | Taiwan Oriental | 91.84% | Iran | 86.26% | Ecuador Amerindian | 77.16% |
| France | 97.87% | Georgia Caucasoid | 91.66% | Brazil Caucasoid | 86.04% | Israel Jew | 77.01% |
| France Caucasoid | 97.87% | Morocco | 91.46% | Brazil | 85.93% | Singapore Oriental | 76.81% |
| United States Caucasoid | 97.45% | Philippines | 90.77% | Venezuela | 85.81% | Mali | 76.76% |
| United States Polynesian | 97.20% | Philippines Austronesian | 90.77% | Australia Australian Aborigines | 85.26% | Mali Black | 76.76% |
| East Asia | 96.94% | Tunisia | 90.75% | Israel Arab | 85.15% | Malaysia Oriental | 75.75% |
| England | 96.66% | Tunisia Arab | 90.74% | Sudan Mixed | 85.09% | Lebanon | 75.41% |
| Korea; South | 96.61% | Chile Amerindian | 90.68% | Sudan | 84.66% | Southwest Asia | 75.13% |
| Korea; South Oriental | 96.61% | Morocco Arab | 90.68% | South Africa | 84.54% | Turkey | 75.10% |
| Japan | 96.30% | Georgia | 90.59% | Venezuela Amerindian | 84.51% | Turkey Caucasoid | 75.10% |
| Japan Oriental | 96.30% | Portugal | 90.46% | Central Africa | 84.23% | Switzerland | 75.00% |
| Finland | 96.29% | Portugal Caucasoid | 90.46% | Cameroon | 84.08% | Switzerland Caucasoid | 75.00% |
| Finland Caucasoid | 96.29% | Austria | 90.44% | Cameroon Black | 84.08% | Jordan | 74.46% |
| Poland | 96.18% | Austria Caucasoid | 90.44% | West Indies | 83.79% | Jordan Arab | 74.46% |
| Poland Caucasoid | 96.18% | Mexico Amerindian | 90.29% | Norway | 83.19% | Senegal | 72.89% |
| Europe | 96.11% | Bulgaria | 90.12% | Norway Caucasoid | 83.19% | Senegal Black | 72.89% |
| Russia | 95.97% | Oceania | 89.98% | Zimbabwe | 82.15% | Ethiopia | 72.41% |
| New Caledonia | 95.80% | United States Black | 89.54% | Zimbabwe Black | 82.15% | Ethiopia Black | 72.41% |
| New Caledonia Melanesian | 95.80% | Australia Caucasoid | 89.43% | South America | 81.83% | Pakistan Asian | 72.34% |
| Russia Siberian | 95.68% | North Africa | 89.18% | American Samoa | 81.53% | Pakistan | 71.73% |
| Czech Republic Caucasoid | 95.46% | South Asia | 89.16% | American Samoa Polynesian | 81.53% | Central African Republic | 70.98% |
| United States | 94.99% | Southeast Asia | 88.97% | Chile Mixed | 81.52% | Central African Republic Black | 70.98% |
| Papua New Guinea | 94.87% | Mexico | 88.96% | Georgia Kurd | 81.33% | Uganda | 70.95% |
| Papua New Guinea Melanesian | 94.87% | United States Amerindian | 88.68% | Mongolia | 81.26% | Uganda Black | 70.95% |
| North America | 94.86% | Northeast Asia | 88.43% | Mongolia Oriental | 81.26% | Mexico Mestizo | 70.29% |
| Czech Republic | 94.84% | China | 88.29% | Guinea-Bissau | 80.45% | Indonesia | 70.23% |
| Sweden | 94.84% | China Oriental | 88.29% | Guinea-Bissau Black | 80.45% | Indonesia Austronesian | 70.23% |
| Sweden Caucasoid | 94.84% | Singapore Austronesian | 88.29% | Denmark | 80.38% |  |  |
| World | 94.41% | Cape Verde | 88.08% | Denmark Caucasoid | 80.38% |  |  |
| United States Asian | 94.11% | Cape Verde Black | 88.08% | Croatia | 80.06% |  |  |
| Saudi Arabia | 93.90% | Chile | 87.93% | Croatia Caucasoid | 80.06% |  |  |
| Saudi Arabia Arab | 93.90% | Scotland | 87.87% | South Africa Other | 79.32% |  |  |
| Russia Caucasoid | 92.99% | Scotland Caucasoid | 87.87% | Bulgaria Other | 79.18% |  |  |
| United States Mestizo | 92.99% | Vietnam | 87.87% | Cuba Mixed | 79.12% |  |  |
| Cuba | 92.60% | Vietnam Oriental | 87.87% | Belgium | 78.93% |  |  |
| United States Hispanic | 92.58% | India | 87.84% | Belgium Caucasoid | 78.93% |  |  |


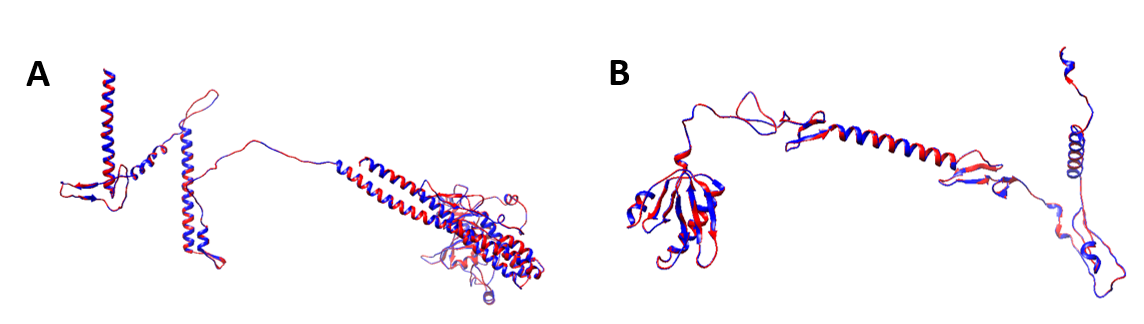


**Figure S2**: Superimposed 3D structures of FAVCs before (blue color) and after (red colour) disulfide bridging for (A) FAVC-FSE and (B) FAVC-CTB


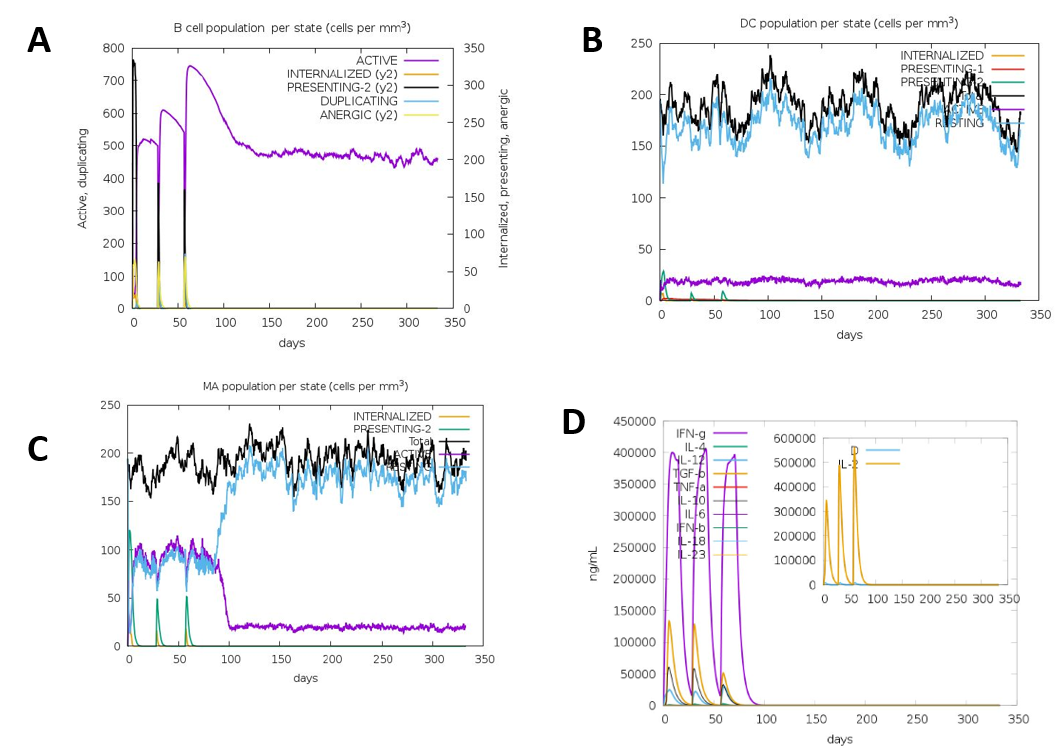
**Figure S3.** The plots for C-IMMSIM immune simulation of the predicted epitopes of FAVC-FSE (A) Active B cell lymphocytes (B) Dendritic cells (C) Macrophages (D) Cytokines and interleukins.

**Figure S4.** The plots for C-IMMSIM immune simulation of the predicted epitopes of FAVC-CTB (A) Active B cell lymphocytes (B) Dendritic cells (C) Macrophages (D) Cytokines and interleukins
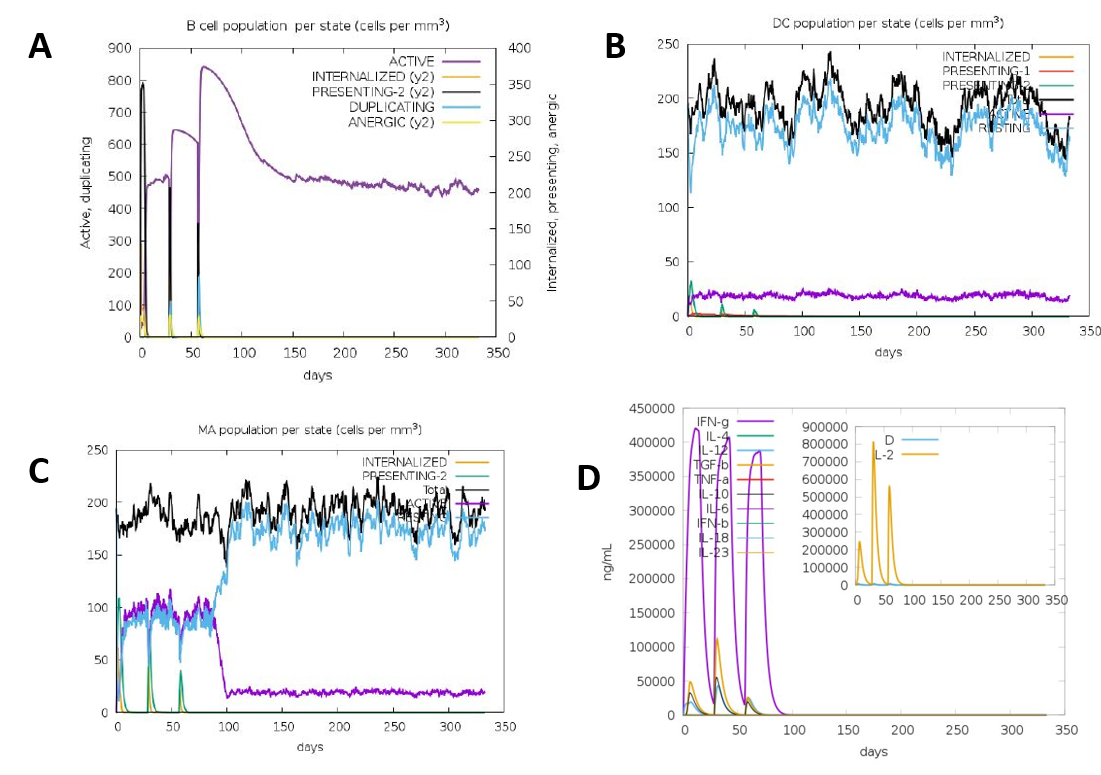


**Table S16**: Amino acid residues of TLR5 used as binding sites which was generated from Naccess 2.1.1 package

| ARG-37 | ILE-35 |
| --- | --- |
| ASN-213 | ILE-99 |
| ASN-265 | LEU-56 |
| ASN-268 | LYS-182 |
| ASN-277 | LYS-242 |
| ASN-31 | LYS-303 |
| ASN-36 | LYS-77 |
| ASP-155 | MET-269 |
| ASP-53 | PHE-180 |
| GLN-129 | PHE-273 |
| GLN-210 | PHE-278 |
| GLN-74 | PHE-75 |
| GLN-80 | SER-26 |
| GLU-214 | SER-271 |
| GLU-327 | SER-272 |
| GLU-79 | SER-55 |
| GLY-270 | THR-208 |
| HIS-275 | TYR-105 |
| ILE-100 | TYR-215 |
| ILE-28 | TYR-267 |
| ILE-33 | TYR-51 |


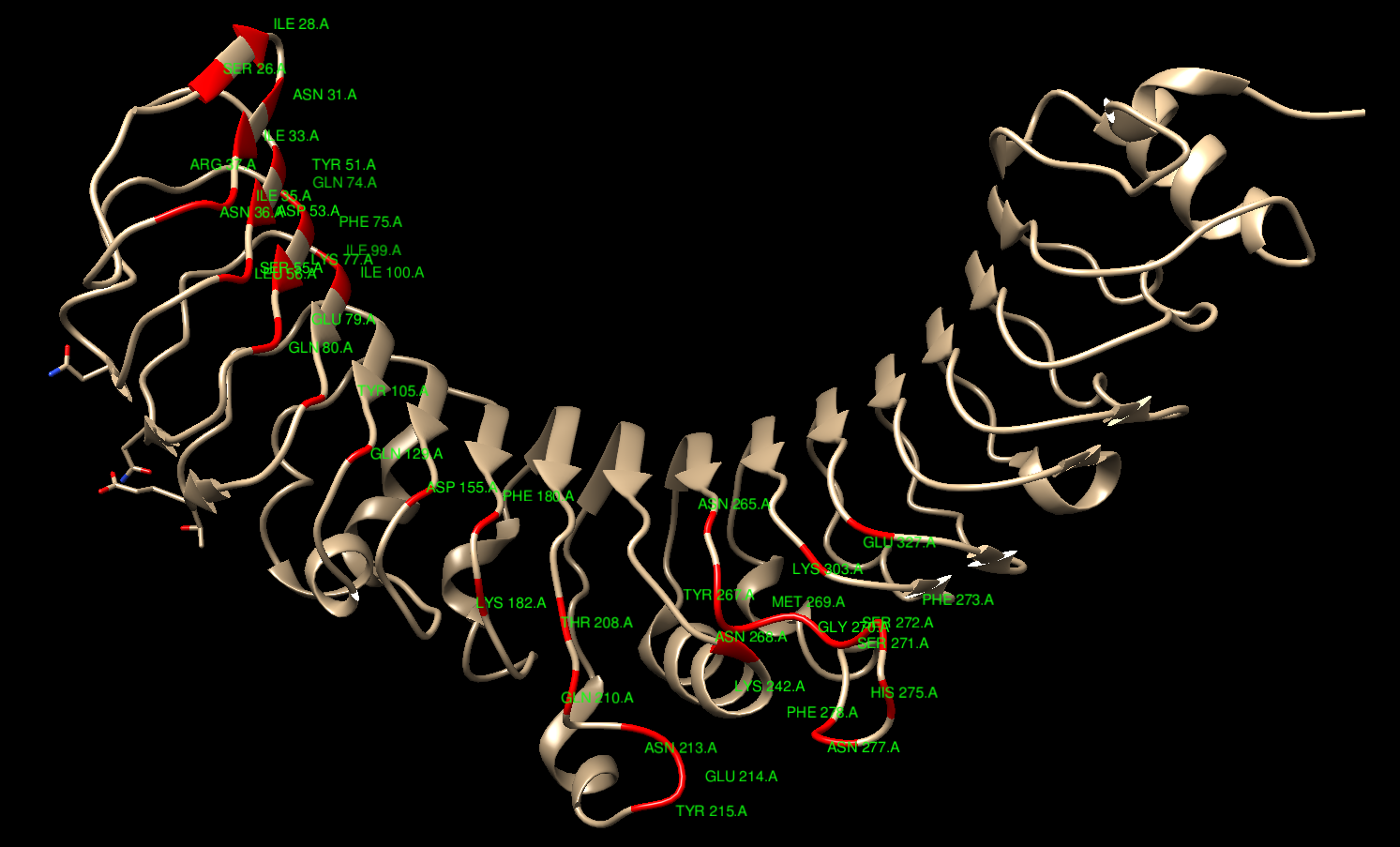


**A**


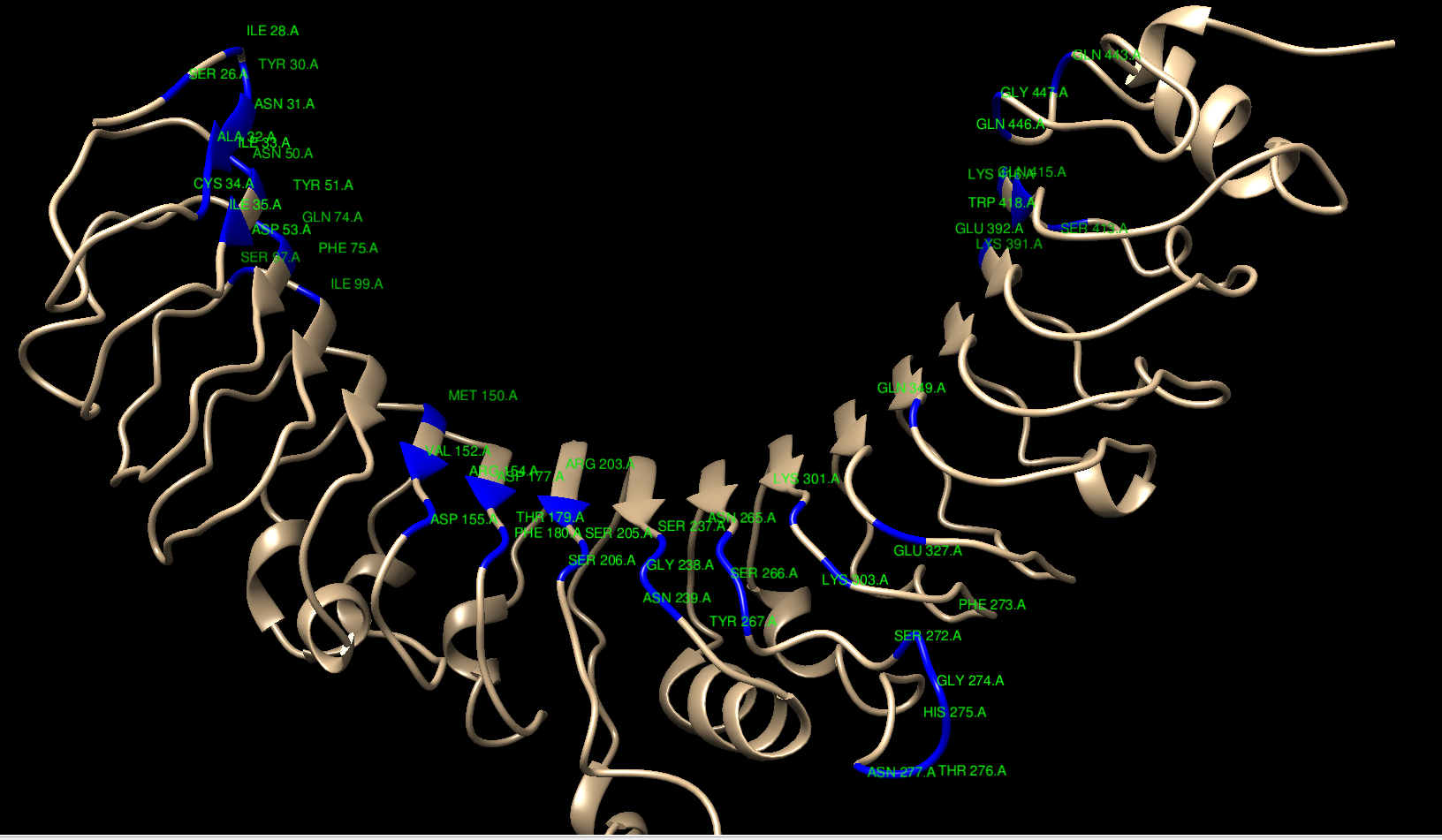


**B**


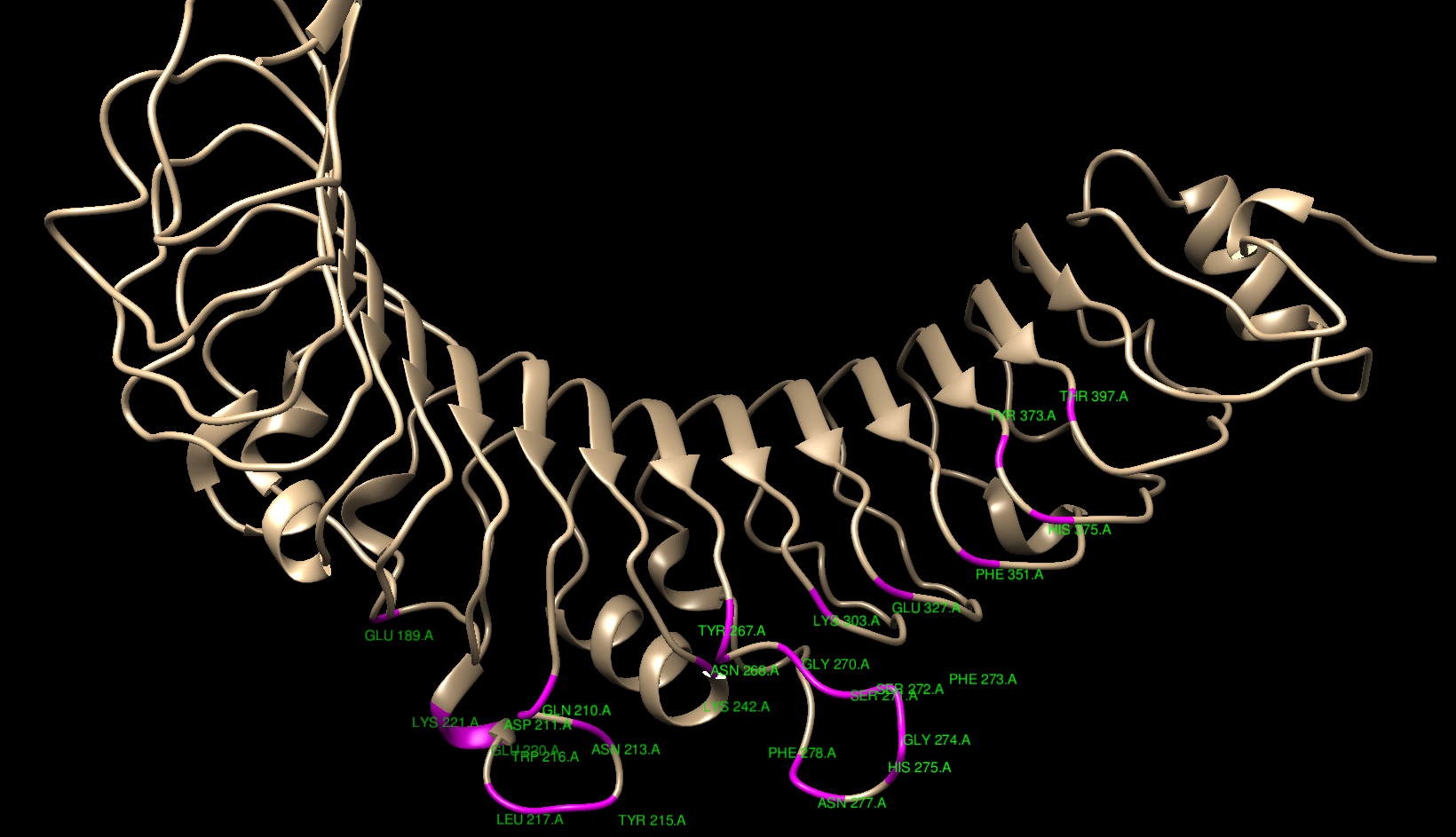


**C**

**Figure S5**: (A) The binding sites of TLR5 with Flagellin *Salmonella* generated from chain A and C of 3V47 downloaded from protein data bank. (B) The binding sites of TLR5 generated after molecular docking with FAVC-FSE. (C) The binding sites of TLR5 generated after molecular docking with FAVC-CTB.

**Table S17.** Decomposed binding residues of FAVC-FSE and FAVC-CTB interacted with TLR5 residues following molecular dynamics simulation with their respective peptide source displayed in Table 1 of the main manuscript. P1 (431-490), P3 (496-555), P5 (561-620), P10 (626-655), EAAAK (linker) (491-495, 556-560, 621-625) for FAVC-FSE. P1 (109-168), P3 (174-233), P5 (239-298), P10 (304-333), EAAAK (linker) (169-173, 234-238, 299-303) for FAVC-CTB

| FAVC-FSE | | FAVC-CTB | |
| --- | --- | --- | --- |
| Interacting FAVC residue | Peptide source | Interacting FAVC residue | Peptide source |
| PHE 507 | P3 | PHE 185 | P3 |
| TYR 511 | P3 | ASN 213 | P3 |
| VAL 546 | P3 | GLU 234 | Linker |
| PRO 451 | P1 | PHE 178 | P3 |
| ILE 548 | P3 | LEU 225 | P3 |
| PHE 500 | P3 | TYR 189 | P3 |
| GLY 544 | P3 | HIS 214 | P3 |
| CYS 443 | P1 | LYS 313 | P10 |
| LEU 503 | P3 | ASN 175 | P3 |
| ASN 549 | P3 | LEU 182 | P3 |
| LEU 469 | P1 | LEU 310 | P10 |
| TYR 499 | P3 | GLU 228 | P3 |
| LYS 486 | P1 | LEU 192 | P3 |
| CYS 487 | P1 | VAL 224 | P3 |
| MET 496 | P3 | LEU 188 | P3 |
| SER 561 | P5 | MET 174 | P3 |
| CYS 467 | P1 | ASN 329 | P10 |
| ILE 447 | P1 |  |  |
| LYS 458 | P1 |  |  |
| ASN 446 | P1 |  |  |
| LEU 504 | P3 |  |  |
| GLN 454 | P3 |  |  |
|  |  |  |  |

TLR5 = Toll-like receptor 5; FAVC = Fused antigen vaccine candidate
